# Remote Follow-Up Technologies in Traumatic Brain Injury: A Scoping Review

**DOI:** 10.1089/neu.2022.0138

**Published:** 2022-09-29

**Authors:** Brandon G. Smith, Stasa Tumpa, Orla Mantle, Charlotte J. Whiffin, Harry Mee, Davi J. Fontoura Solla, Wellingson S. Paiva, Virginia F.J. Newcombe, Angelos G. Kolias, Peter J. Hutchinson

**Affiliations:** ^1^ Division of Neurosurgery, Department of Clinical Neurosciences, Addenbrooke's Hospital and University of Cambridge, Cambridge, United Kingdom.; ^2^ NIHR Global Health Research Group on Neurotrauma, University of Cambridge, Cambridge, United Kingdom.; ^3^ School of Clinical Medicine, University of Cambridge, Cambridge, United Kingdom.; ^4^ GKT School of Medical Education, King's College London, London, United Kingdom.; ^5^ College of Health, Psychology and Social Care, University of Derby, Derby, United Kingdom.; ^6^ Division of Rehabilitation Medicine, Addenbrooke's Hospital and University of Cambridge, Cambridge, United Kingdom.; ^7^ Division of Neurosurgery, Department of Neurology, University of São Paulo, São Paulo, Brazil.; ^8^ University Division of Anesthesia, University of Cambridge, Cambridge, United Kingdom.

**Keywords:** follow-up technology, innovation, outcome assessment, patient-generated health data, telemedicine, traumatic brain injury

## Abstract

Traumatic brain injury (TBI) remains a leading cause of death and disability worldwide. Motivations for outcome data collection in TBI are threefold: to improve patient outcomes, to facilitate research, and to provide the means and methods for wider injury surveillance. Such data play a pivotal role in population health, and ways to increase the reliability of data collection following TBI should be pursued. As a result, technology-aided follow-up of patients with neurotrauma is on the rise; there is, therefore, a need to describe how such technologies have been used. A scoping review was conducted and reported using the PRISMA extension (PRISMA-ScR). Five electronic databases (Embase, MEDLINE, Global Health, PsycInfo, and Scopus) were searched systematically using keywords derived from the concepts of “telemedicine,” “TBI,” “outcome assessment,” and “patient-generated health data.” Forty studies described follow-up technologies (FUTs) utilizing telephones (52.5%, *n =* 21), short message service (SMS; 10%, *n =* 4), smartphones (22.5%, *n =* 9), videoconferencing (10%, *n =* 4), digital assistants (2.5%, *n =* 1), and custom devices (2.5%, *n =* 1) among cohorts of patients with TBI of varying injury severity. Where reported, clinical facilitators, remote follow-up timing and intervals between sessions, synchronicity of follow-up instances, proxy involvement, outcome measures utilized, and technology evaluation efforts are described. FUTs can aid more temporally sensitive assessments and capture fluctuating sequelae, a benefit of particular relevance to TBI cohorts. However, the evidence base surrounding FUTs remains in its infancy, particularly with respect to large samples, low- and middle-income patient cohorts, and the validation of outcome measures for deployment via such remote technology.

## Introduction

Traumatic brain injury (TBI) is a concern in both public and global health, and is a leading cause of death and disability worldwide.^1,2^ Secondary deficits from TBI manifest in multiple ways, often with long-term symptoms in physical, cognitive, and emotional domains,^3–5^ which have a collateral impact, both direct and indirect, on patients, families, and wider society.^2,3,6,7^ The purpose of outcome data collection is threefold: to improve patient outcomes, to facilitate research, and to provide the means and methods for wider injury surveillance.

Each purpose may vary in the fidelity of the data sought—injury surveillance efforts may typically aim to garner general morbidity or mortality on a wider scale, whereas research initiatives may use refined, detailed assessment batteries centered upon the phenomena or sequelae in question. In clinical practice, we may seek a balance of both—employing more detailed outcome measures and assessments as required, while also addressing any ongoing sequelae and determining the general status and well-being of the patient.

In 2004, the World Health Organization (WHO) released Guidelines for Essential Trauma Care emphasizing the importance of surveillance data to reduce the global burden of death and disability from injuries.^8^ In addition to enabling clinical teams to determine the full extent of physical, mental, and socioeconomic sequalae post-injury,^5,9–11^ accurate data also facilitate the evaluation of systems and services, including: evaluating the efficacy of patient treatment and management decisions^10,11^; identifying targets of wider systems improvement in injury prevention^12^; enabling continuous quality improvement projects and trials; enabling the formation of registries that may themselves be incorporated into care pathways, injury prevention strategies, and policies^13,14^; and lastly, facilitating rehabilitation of an individual and improving rehabilitation pathways and services.

Despite improvements in injury surveillance data, data on disability and long-term functional outcomes remain poorly recorded in both high-income countries (HICs) and low- and middle-income countries (LMICs).^5,10,15,16^ However, given the heterogeneity both within and between LMICs, the collection of outcome data is considered more complex^1,10,17^ and as such is often limited to collection at hospital discharge only.^10^ Despite these limitations very little has been published on the challenges faced in facilitating long-term follow-up and collection of outcome data in LMICs. Of the research that does exists, efforts in data collection at the clinical level were found to be complicated by factors such as weak health care and long-term support infrastructure, resulting in a lack of regular follow-up of patients with trauma.^10^

In 2007 the world's population became more urban than rural for the first time.^18^ However, in many countries, and in particular LMICs, vast numbers remain in rural settings, and with this, have limited access to general health services. In the context of specialist services such as neurosurgery, which remain heavily centralized to urban settings, clinicians often have limited or no regular access to patients after discharge.^19–21^ Patients who are able to access neurosurgical follow-up often have to travel extensive distances at great personal cost.^22,23^ Those unable to access neurosurgical follow-up become lost to follow-up.^24,25^

Over the last few decades, there has been rapid advancements in technology, especially regarding telecommunications and its widespread adoption. According to a United Nations (UN) International Telecommunication Union (ITU) 2020 report,^26^ 47% of households worldwide are estimated to have access to a computer, with 57% of households perceived to have internet access. Mobile phone and cellular network technologies, however, are arguably the “common-denominator” technology worldwide. An estimated 75 per 100 of the world's population are thought to have an active mobile broadband connection, and it is estimated that there are 105 mobile-cellular telephone subscriptions per 100 population.

Similarly, short message service (SMS) technologies, initially made possible by second-generation or “2G” cellular network technology, remains a valuable opportunity for outcome data collection owing to its worldwide penetration and strong coverage, even in an LMIC setting where it is estimated that over 95% of the population have 2G coverage.^27^ The adoption of next-generation network architecture, such as fifth generation (5G) cellular technology, can be expected to lead to exciting new possibilities for mobile health assessment owing to its ability to provide accessible, high-speed streaming capabilities^28^ for use in high-definition remote video assessments.

Technologies are becoming frequently adapted to harness unique opportunities to connect patients with their health providers upon discharge. These follow-up technologies (FUTs) may provide innovative solutions that mitigate those deemed “lost to follow-up,” fill quantitative gaps in TBI epidemiology, and enable those in the remotest corners of the world to access specialist care.

Prior engagement with the literature on FUTs revealed a complex and diverse evidence base, and no prior attempt to synthesize this substantial body of work. A scoping review was therefore deemed an appropriate methodology to describe the type, characteristics, and size of the evidence in this field. The objective of this review was to identify follow-up technologies, such as telephone- and SMS-based service, videoconferencing, and smartphone applications, implemented across global settings in TBI. In addition, this review aimed to characterize the outcome measures administered and the data collected, among the communication modalities used, and briefly highlight the success of each in context with the patient populations and settings they have been implemented in.

## Methods

**FIG. 1.**
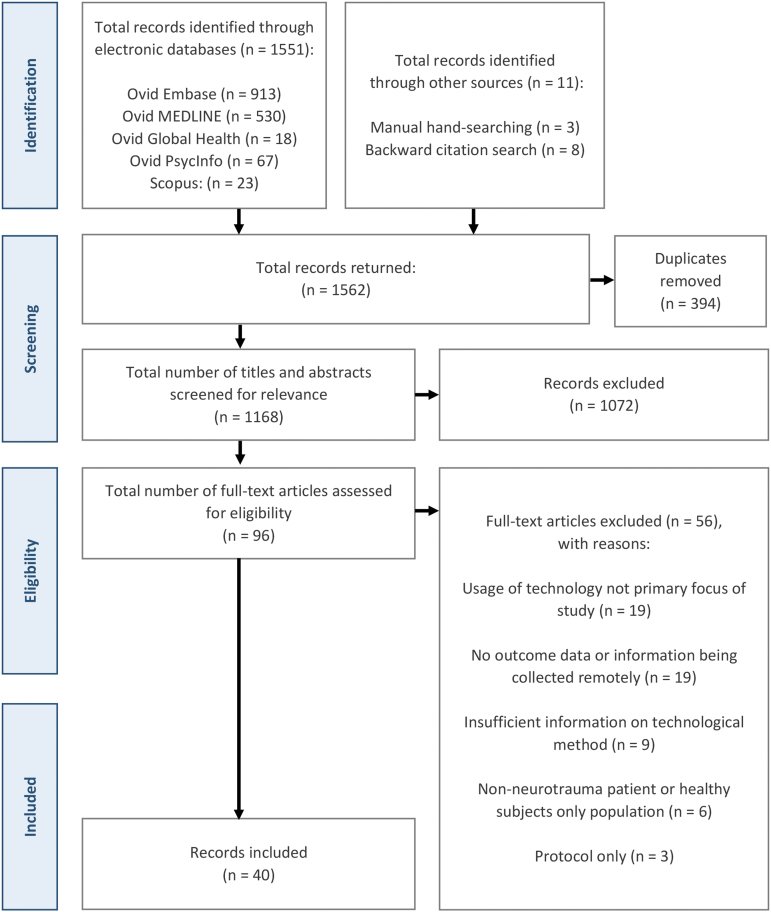
Preferred Reporting Items for Systematic Reviews and Meta-Analyses (PRISMA) flowchart detailing the study selection process.

The reporting of this study is in accordance with the Preferred Reporting Items for Systematic Reviews and Meta-Analyses extension for scoping reviews (PRISMA-ScR),^29^ with an additional study screening and selection flowchart (Fig. 1) as recommended by the Joanna Briggs Institute (JBI) and PRISMA-ScR reporting guidelines.^29–31^ Unlike systematic reviews, scoping reviews do not require an antecedent protocol registration,^32^ notwithstanding, review objectives, eligibility criteria, and preliminary study characteristics to be extracted were determined *a priori.*

Our scoping review was guided by the comprehensive scoping review framework by Arksey and O'Malley,^33^ with minor refinements, including those suggested by Levac and colleagues^34^ and the JBI.^30^ This framework includes: (1) identifying the research question; (2) identifying relevant studies; (3) study selection; (4) charting the data; (5) collating, summarizing, and reporting the results; and an optional (6) consultation exercise. This review serves as part of a wider program of work in which stakeholders (neurotrauma physicians) are formally engaged in a separate qualitative research process, examining their views, experiences, and opinions of FUTs as applied to post-TBI care provision. Prior to commencing the review, we shared similar perceptions to those of Levac and colleagues^34^ regarding the challenges of conducting and integrating the results of stakeholder consultation within a review's findings. In light of this, and given the potential of qualitative inquiry to empower rich and in-depth investigations of the human experience,^35^ stage 6 was not carried out within this review. Notwithstanding, the lead author (BGS) discussed findings with senior co-authors, who by their nature as LMIC neurosurgeons are stakeholders within this context.

### Research question

What research has been conducted to describe, examine, or assess the use of follow-up technologies in traumatic brain injury cohorts worldwide?

To answer this question, the following sub-questions were posed:

1. What technologies are being used as FUTs in a global setting?
2. In what settings is FUT research being conducted?
3. What patient cohorts (demographics, injury severity) have been included in FUT research?
4. What factors may constitute successful implementation of FUTs?
5. What, if any, validated outcome measures are being deployed via FUTs?

### Search strategy

The final search strategy was determined with the assistance of an academic medical librarian following a consultation to derive keywords based on the review objective and concepts of “telemedicine,” “traumatic brain injury,” “outcome assessment,” and “patient-generated health data.” The authors selected a number of “indicator papers”—predefined articles that one would expect to appear in their final search results—to test the quality and robustness of the search strategy. Several pilot searches were attempted before a final strategy was established and translated across a number of databases. The search strategy was executed on the October 1, 2021 on the following electronic databases: OVID Embase, OVID MEDLINE, OVID Global Health, OVID PsycInfo, and Scopus. An example search strategy for OVID MEDLINE can be found in Supplementary Table S2. These databases were selected owing to their sufficient coverage given the multi-disciplinary nature of TBI, outcomes, and their assessment. A limited manual search was conducted on Google Scholar and a number of technology- and head-injury focused journals (*Journal of Neurotrauma, The Journal of Head Trauma Rehabilitation, World Neurosurgery, NEUROSURGERY, Journal of Medical Internet Research, Journal of Telemedicine and Telecare*).

Following a pre-protocol pilot gray literature search, it became apparent that, of the limited materials retrieved, most were unsuitable for a number of reasons, including: insufficient indication of external peer review (of particular importance in commercial reports); a lack of FUT description or elucidation of the methods of their use; and description or evaluation of the FUT was not perceived to be the primary focus of the resource. Compounded by the resource- and time-intensive nature of conducting a gray literature search in this context, gray literature sources were omitted in the final search strategy. Finally, backward citation searching was undertaken, whereby the reference lists of articles deemed eligible for inclusion, and review articles that were not eligible for inclusion, were screened for relevant studies. Searching for additional sources was completed on April 25, 2022.

### Eligibility criteria

Scientific articles reporting original research of the application of technology written in English were included. All databases were searched from inception to achieve the largest scope possible and to detail early innovations in this field.

The Population/Participants, Concept & Context (PCC) framework^36,37^ was used to inform our inclusion criteria, search, and data charting strategies.

Eligibility criteria for articles to be included in this review were: (1) any published original research, including: primary studies, reports, editorials, opinion articles, letters, conference abstracts, theses, and book chapters; (2) reports with a primary aim to describe, assess, or examine the use of FUTs to facilitate remote collection of patient outcome data; and (3) adult and pediatric all-severity cohorts of TBI patients. Articles were excluded if they were (1) study protocols or secondary research (reviews); (2) reports describing the collection of family or caregiver outcomes only; or (3) no TBI population or involved healthy volunteers only.

### Population/Participants

Studies were only eligible for inclusion in this review if their primary aim related to the development, implementation, or validation of technologies contributing to the provision of follow-up care of discharged patients following TBI of any severity, whether directly or via proxy (family members/relatives, caregivers, and guardians). Studies of mixed-pathology cohorts were included.

### Concept

In this review, we defined follow-up technology as any system, device, equipment, component, or machinery used to both transmit and receive digital information electronically between a remote outpatient or their proxy and a member of their clinical team. The focus of this exchange was to attain data from the patient as to their current welfare status in the form of either patient-generated health data (PGHD), that is, self-reported, or clinician-derived health data (CDHD), that is, garnered through clinician-led assessment or interview, either from the patient or their nominated proxy. In addition, we defined “remote” as, at the time of information exchange, the patient was in a community-based setting (e.g., home, regional care provider such as a general practitioner or regional hospital, or other public settings) at a distance from the clinical team managing their follow-up.

Herein, we refer to synchronicity as the temporal aspect of the encounter between clinician and patient. Synchronous FUTs function to collect data in real time, often allowing direct contact between clinician and patient through sensor-, text-, voice-, or video-based technology. Asynchronous FUTs collect data by store-and-forward techniques—data are gathered, stored, and transmitted for later review by the clinician at two independent time-points; that is, they do not interact in real time.^38^

Finally, we define “follow-up” as any attempt to monitor, assess, communicate, or liaise with a patient, or their proxy, from the point of hospital discharge, for the benefit of furthering their health and well-being, research, or injury surveillance.

### Context

#### FUTs utilized in any global health setting

We utilized HIC and LMIC classifications as defined by the 2021–2022 World Bank list of economies.^39^

### Study selection

A two-stage screening process was followed. All search results were initially imported into the Zotero (Corporation for Digital Scholarship, Virginia, USA) reference manager for title and abstract review. Where necessary, duplicates were removed manually. Two researchers (BGS and ST) independently screened all titles and abstracts, and potentially eligible studies were identified for full-text review.

Disagreements arising from the selection process were either resolved by consensus, or where this was not possible, a third reviewer (OM) was consulted for resolution. Following preliminary screening, the remaining articles were exported to Microsoft^®^ Excel (Microsoft, Redmond, Washington, USA), where full texts were independently screened by two researchers (BGS and ST) for final eligibility; a third researcher (OM) was consulted for disagreements as required. Selected studies formed the final repository of evidence for subsequent data extraction (charting) and collation.

### Data charting and synthesis of results

Owing to the exploratory nature of a scoping review, a precursor proforma was developed to facilitate data extraction. Within this document, initial elements of interest that sought to answer our research question were informed by our PCC framework and agreed upon by researcher consensus (BGS, ST, CJW, AGK). The proforma was subdivided into five key sections, including: authorship and study characteristics, description of neurotrauma, characteristics of FUT(s) utilized, and major findings and challenges. This was iteratively updated and refined as the charting process progressed, adding to pre-identified elements of interest (see Supplementary Table S1). Data charting was conducted independently for all articles by two researchers (BGS and ST). Following the charting of the first five studies, in line with recommendations by Levac and colleagues,^34^ the authors reconvened to ensure proforma suitability in addressing the research question, and to advance the proforma following familiarization with this initial subset of studies. Final completed proformas were cross-checked for conformity, and a third researcher (OM) was consulted as necessary in cases of a dispute. No formal critical appraisal, or quality of evidence assessment, was conducted as it would fall beyond the remit of a scoping review.^40^ Following data charting, a narrative summary of included articles was constructed in relation to the review's overarching question and sub-questions.

## Results

### Characteristics of included studies

Executing the search strategy across the five electronic databases yielded a total of 1562 potentially eligible citations. Following de-duplication, 1168 unique articles remained, with a subsequent title and abstract review delineating a pool of 96 citations for further full-text review. This final stage of screening concluded with 40 articles for inclusion; inclusive of 11 citations discovered through manual and citation searches. A full PRISMA-ScR flowchart for the study search, selection, and exclusion process is depicted in Figure 1.

Of the 40 articles retrieved, the plurality (*n =* 15) were reported or deemed to be descriptive in design,^19,41–54^ and included small, non-randomized pilot studies and secondary analyses of data, or in two studies, were conducted in or described the retrospective analysis of a quality improvement initiative format.^52,53^ Citations with an observational design formed the second most common type (*n =* 10),^10,55–63^ encompassing prospective^55,56,58^ and retrospective^10^ cohort studies and cross-sectional studies.^57,60,61^ Research of experimental (n = 9)^64–72^ and quasi-experimental (*n =* 6)^73–78^ design was similar in frequency. Experimental designs included single-center^66,68–71^ and international multi-center^65,67^ randomized trial designs, among non-randomized, open-label trials.^64,72^ Results of these studies were published primarily as original research articles (*n =* 31),^10,19,41–44,48–53,55,56,59,61–75,78^ with a lesser number as conference abstracts or research posters (*n =* 8),^45–47,57,58,60,76,77^ and research summary letters (*n =* 1).^54^

### International context

The adaption of technologies for follow-up delivery has been used in multiple settings throughout the world. The majority of articles reported on FUTs in HICs (*n =* 34, 85%), including the United States (*n =* 24),^41–43,51–61,63,64,66,67,72–74,76–78^ Australia (*n =* 3),^70,71,75^ Canada (*n =* 3),^45,47,68^ the Netherlands (*n =* 1),^62^ Ireland (*n =* 2)^46,50^ and a joint endeavor between Italy, Spain, and Belgium (*n =* 1).^65^ Whereas only six (15%) reported findings from studies in LMICs including Uganda (*n =* 2),^19,49^ Ethiopia (*n =* 1),^10^ India (*n =* 1),^44^ Iran (*n =* 1),^69^ and Indonesia (*n =* 1).^48,79^

### Patient population demographics and TBI characteristics

The majority of articles (*n =* 27, 67.5%) described civilian adult population cohorts (>18 years of age),^10,41,42,44–48,50–52,55,57–60,62,65,66,69–71,73–75,77,79^ from sample statistics reported representing 3442 patients. A further five studies described military or veteran cohorts^43,61,64,67,76^ representing an additional 207 adult patients. Five studies reported exclusively pediatric patient cohorts,^19,54,63,68,72^ representing 287 patients. Three studies investigated mixed adult and pediatric cohorts,^49,56,78^ adding a further 774 patients to previous approximations. Two studies did not report the demographics of the cohorts investigated.^44,53^

With respect to TBI severity, FUTs were most frequently implemented in cohorts of patients with TBI of undefined severity (*n =* 14)^10,44,45,49,52,53,55,58,62,65,67,70,71,77^—in some of these cases, patient cohorts were pooled with other diagnoses (trauma, spinal cord injury, stroke, orthopedic, acquired brain injury, among other neurological conditions). Where TBI severity was defined, seven studies (*n =* 7) explored the implementation of FUTs in all-severity TBI patient cohorts.^19,41,48,51,73,74,76^ In studies recruiting patients with particular injury severities, concussion or mild TBI formed the majority (*n =* 12),^43,46,50,54,56,59,61,63,66,68,72,78^ whereas only one study investigated FUTs as applied to a cohort of patients with severe TBI exclusively.^75^ No studies reported moderate TBI cohorts exclusively. Of the remaining studies, two reported FUTs for mild to moderate TBI cohorts,^47,69^ and four for moderate to severe TBI cohorts.^42,57,60,64^ Further, two studies described their TBI cohort as chronic.^51,58^

Almost all (*n =* 37) studies used follow-up technologies while the patient was at home or in another non-health setting in the community. The remaining studies (*n =* 3) used technology while the patient was at other clinical settings away from the team responsible for follow-up assessment, including a polytrauma rehabilitation center in one study,^76^ and a hospital research laboratory 15 km from the assessing clinician^70,71^ in two linked studies by the same author team.

### Technology modality

**FIG. 2.**
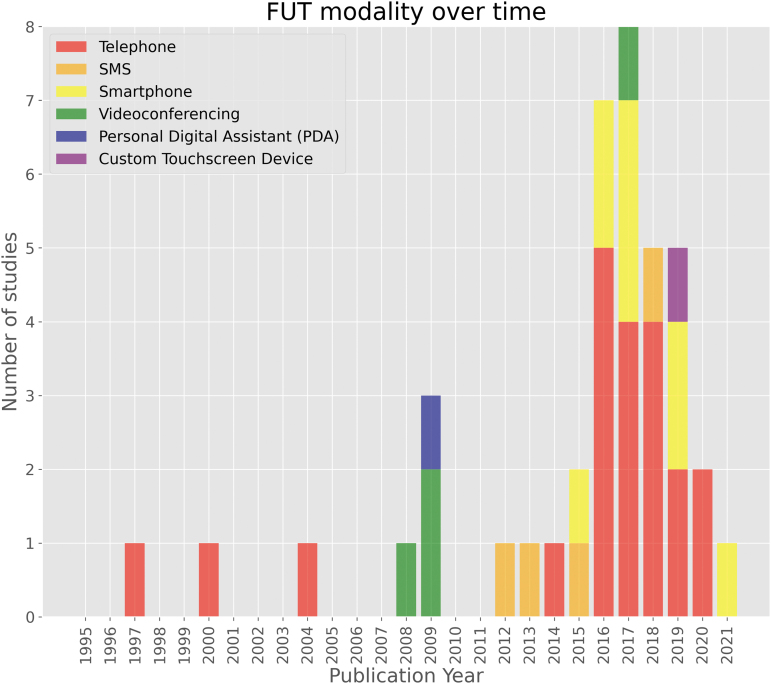
Follow-up technology modality over time. PDA, personal digital assistant; SMS, short message service.

Remote FUTs were grouped by their underlying modality: telephone, SMS, smartphone (e.g., mobile application), videoconference, and “miscellaneous”—namely a personal digital assistant (PDA) and custom touchscreen device—each demonstrated in one study respectively. Telephone-based FUTs (52.5%) were the first to appear for use with cohorts of patients with TBI in 1997,^41^ and remained the modal technology utilized at the time of search strategy execution. Videoconference-based technologies (10%) were next to appear a decade later in 2008 as part of a multi-center randomized trial.^65^ A single study exploring PDA (2.5%) as a remote FUT for ecological momentary assessment (EMA) in a pediatric concussion cohort followed shortly after in 2009.^63^ SMS-based FUTs (10%) followed in 2012, forming the asynchronous communication element of a pilot EMA study in the United States.^43^ Smartphone-based FUTs (22.5%) were introduced in 2015. Lastly, a single study^62^ explored the use of a custom electronic touchscreen device, the PsyMate, in 2019 to investigate the feasibility of EMA to explore the interactions between person, environment, and effect in an acquired brain injury (ABI) cohort. Figure 2 demonstrates the technology modalities implemented over time.

### Telephone-based follow-up

Telephone-based follow-up technology (Table 1) was used in 21 studies (52.5%).^10,19,41,42,44–50,52,53,55,57,60,64,68,69,74,76^ Telephones were primarily used as a synchronous means of two-way communication to conduct structured interviews with patients and their proxies. Although almost all studies, where defined, used clinical or research staff to conduct follow-up, one used an external call center that had integrated its systems with the hospital's electronic medical records^44^ to complete the follow-up interview. Another study^55^ had no human facilitator in the administration of telephone-based follow-up, and instead used an asynchronous and interactive voice response system (IVR), whereby pre-recorded questions were played to the patient, and either a voice or keypad could be used to respond. One study that used scripted, structured telephonic follow-up described the additional use of a secure web-based data capture platform (REDCap) with branching logic to conduct the interview.^52^ Lastly, one study reported the use of a computer-assisted telephone interviewing system, whereby the assessor could follow a script in the collection of data, enabling an assessor without familiarity with TBI to conduct the interview.^74^

**Table 1.** Citations Reporting the Use of Telephone-Based Follow-Up

Author, year (country)	Study design (author definition)	Study aim/Objective	Sample Population demographics TBI characteristics	Follow-up technology (FUT) description Clinical facilitator Sessions & instances count	Synchronicity Use of proxy	Constructs & outcome measures deployed	Response/Success/Compliance rates
Dombovy et al., 1997 (USA)^41^	Descriptive	To determine if functional, neuropsychological, and social outcome at 3 and 6 months in patients hospitalized following traumatic brain injury (TBI) could be ascertained via telephone follow-up, and assess use of rehabilitation services in this population.	*n =* 74 adult TBI patients (all-severity) at home/community Average age (SD) = 39.2y Sex (F) = 29.7% Mean admission GCS = 11.5 Mild or moderate = 77% Severe = 23% TSI/D = 3 months ±2 weeks	Telephone-based assessment at 3- and 6-months post-injury Nurse practitioner 2 sessions, 15–30 min in duration	Synchronous No reported use of proxy	Functional Independence Measure (FIM) Telephone Interview for Cognitive Status (TICS) Neurobehavioral Rating Scale (NRS) Generic questions regarding employment, household activities, personal finance management, travel & social activities	Telephone deemed a cost-effective way to ascertain functional and neuropsychological outcomes in TBI survivors, and may identify those who may benefit from additional rehabilitation
**Warden et al., 2000 (USA)^64^**	Experimental (non-randomized, open label, controlled)	To compare home versus inpatient cognitive rehabilitation for patients with moderate to severe head injury	*n =* 53 adult (military) TBI patients (moderate-severe) at home/community in home-program arm of trial Average age (SD) = 26y (6.22) Sex (F) = 4% Mean admission GCS = 9.5 TSI/D (SD) = 39 days (33.2)	Telephone-based support (information providing, problem solving, support and encouragement) and assessment Psychiatric nurse Weekly sessions over 8 weeks	Synchronous Family members able to contact nurse as required	Weekly generalized overall well-being checklist (headache, irritability, fatigue, depression, memory problems, medication compliance, miscellaneous problems requiring intervention) Activities at week 2 and 7 (shopping, watching television, community activities, and socializing with friends)	*n =* 47 (88.7%) completed the telephone-based program
**Bell et al., 2004 (USA)^42^**	Descriptive	To describe the development of a telephone follow-up program that addresses the needs of survivors of TBI and their families in the year following injury	*n =* 84 adult (moderate-severe) TBI patients at home/community Average age (SD) = 34.4y (13.6) Sex (F) = 18% Moderate to severe TSI/D = 2 weeks following discharge	Telephone-based assessment interview and provision of support and information Research care manager 7 planned contacts at 2 weeks, 4 weeks, 2 months, 3 months, 5 months, 7 months, 9 months	Synchronous Use of proxy at each contact (family member or significant other)	Non-specific review of past and current concerns with triage/referral as required At 4 weeks and 9 months, structured interview addressing 17 domains: personal care, ambulation, travel, work, school, home management, leisure, social integration, cognitive and behavioral concerns, standard of living, financial independence, medical concerns, emotional function, alcohol use, drug use, legal issues, and spirituality	Median 4 contacts with patients and 4.5 with relatives over 9-month period Median call duration 34 min with TBI patients, 30 min with relatives
**Wong et al., 2014 (USA)^55^**	Observational (prospective cohort study)	To evaluate the feasibility of computer adaptive testing (CAT) using an Internet or telephone interface to collect patient-reported outcomes after inpatient rehabilitation and to examine patient characteristics associated with completion of the CAT-administered measure and mode of administration	*n =* 674 (mixed diagnosis: spinal cord injury, stroke, orthopedic, “other neurological” or other conditions), of which *n =* 40 adult brain injury patients at home/community Total cohort: average age (SD) = 62.9y (15.7) Sex (F) = 59.8% Not defined	Telephone-based (interactive voice response) or secure Internet website-based self-reported computerized adaptive testing Hospital outcome management department staff One instance of delivery a month following discharge	Asynchronous No reported use of proxy	Community Participation Indicators (CPI) modified for computer adaptive testing (CAT) delivery	Across the diagnosis cohorts, 61.0% chose telephone IVR versus 39.0% chose Internet-based assessment Patients with brain injury had an 81% reduced likelihood of competing any form of assessment with respect to other patient cohorts
**Xavier et al., 2016 (India)^44^**	Descriptive	To assess the Glasgow Coma Outcome Scale via a call center for head-injured patients who were discharged after head injury	*n =* 484 TBI patients at home/community Not reported Not reported	External call center with integration of electronic medical records system to administer structured telephonic questionnaire interview to discharged patients Call center staff Single instance of administration following discharge	Synchronous No reported use of proxy	Glasgow Outcome Scale (GOS) in Hindi	84% (*n =* 406) patients could be reached by phone and GOS elicited 63% of patients were from rural areas
**Mortenson et al., 2016 (Canada)^68^**	Experimental (pilot RCT)	To investigate the effectiveness and feasibility of early intervention telephone counselling with parents in limiting post-concussion symptoms and impacts on children and youth.	*n =* 66 pediatric patients with concussion injury and their parents at home/community (*n =* 32 intervention, *n =* 34 control) Average age (range) = 11.9y (6.3-16.5)[I], 12.6 (5.2-16.8) [C] Sex (F) = 25% [I], 35% [C] Clinically diagnosed mild TBI or concussion, defined as GCS 13/14 at admission or LoC <30 min or altered mental status at injury or post-traumatic amnesia <24 h duration TSI/D = within 1 week of injury	Structured telephone-based follow-up and symptom counseling for parents with children with ongoing symptoms Occupational therapist Two instances at 1-week and 1-month post-injury in intervention group Intervention and control received telephonic Post-Concussion Symptom Inventory (PCSI) at 3 months post-injury	Synchronous Full use of proxy; no patients directly communicated with	Intervention arm at 1 week/1 month: Acute Concussion Evaluation (ACE) Protocol Generalized interview regarding impact of symptoms on everyday function, home, educational and play activities Both groups at 3 months: PCSI Parent Assessment Form	Across both groups, *n =* 8 children were identified to have ongoing symptoms not otherwise identified by regular care at 3 months post-injury No significant difference of post-concussion symptoms between groups at 3 months post-injury *n =* 8 lost to attrition (loss of contact) across both groups from initial randomization of *n =* 76 patients
**Cuthbert et al., 2016 (USA)^74^**	Quasi-experimental (test-retest of stratified random sample from larger primary study)	To provide test-retest reliability (>5 months) of the Ohio State University Traumatic Brain Injury Identification Method modified for use as a computer-assisted telephone interview (CATI) to capture TBI and other substantial bodily injuries among a representative sample of non-institutionalized adults living in Colorado	*n =* 200 adult patients (*n =* 50 TBI with LoC, *n =* 50 TBI w/o LoC, *n =* 50 major lifetime injury w/o TBI, *n =* 50 no major lifetime injury) at home/community *n =* 194: Median age group (range) = 50-59y (18-89). Sex (F) = 30.9% All-severity TBI TSI/D (*n =* 194) = <5 y since injury *n =* 26, 5 < y since injury *n =* 168	Standardized, computer-assisted telephone interview (CATI) Interviewer (undefined) without familiarity with TBI One instance of telephone delivery 6 to 18 months following initial interview	Synchronous No reported use of proxy	Ohio State University TBI Identification Method (OSU TBI-ID)	100% (*n =* 194) instances of CATI completed across 18 months (range 6–15 months) Mean time between initial and telephone follow-up interviews = 10.96 months (SD 2.37) High response and completion rate reported, with 68.3% of patients reached within 5 contact attempts Average delivery time = 5 min (range 1–18 min)
**Varner et al., 2016 (Canada)^45^**	Descriptive (secondary data analysis of RCT data)	To determine if text messaging study participants involved in an ongoing randomized trial resulted in a lower rate of attrition as compared with conventional telephone follow-up	*n =* 118 (*n =* 40 intervention, *n =* 78 control) adult head-injury patients at home/community Average age (SD) = 35.2y (13.7) Sex (F) = 63.6% Presenting ED complaint of “head injury” not otherwise quantified/defined TSI/D = 2–4 weeks post-ED discharge	Telephone-based symptom questionnaire with (intervention)/without (control) prior SMS reminder No defined facilitator Two instances of telephone follow-up at 2 and 4 weeks post-ED discharge	Synchronous No reported use of proxy	Unspecified general symptom questionnaire	*n =* 3 withdrew from control Of *n =* 75 receiving telephone follow-up w/o reminder, 32% at 2 weeks and 42.7% at 4 weeks were unable to be contacted Of *n =* 40 with SMS reminder prior to telephone follow-up, 10% at 2 weeks and 25% at 4 weeks were unable to be contacted
**Deasy et al., 2016 (Ireland)^46^**	Descriptive	To investigates the prevalence of Post-Concussion Syndrome (PCS) and the quality-of-life of patients who were treated in the Clinical Decision Unit (CDU) of Cork University Hospital (CUH) in 2013	*n =* 112 adult TBI patients at home/community Not reported Mild TBI confirmed by records and/or CT imaging review TSI/D = **a**pproximately within 1-year post-injury	Structured telephone-based follow-up No defined facilitator One instance of follow-up within 1-year post-injury	Synchronous No reported use of proxy	Rivermead Post-Concussion Symptoms Questionnaire (RPQ) Quality of Life by 12-Item Short Form Survey (SF-12) Functional outcome (EQ5D5L)	Significant loss to follow-up with only 50.9% able to be reached within 1 year post injury
**Thibault-Halman et al., 2017 (Canada)^47^**	Descriptive	To examine the frequency and severity of common post-TBI symptoms, as assessed by the RPCQ	*n =* 46 adult TBI patients at home/community Not reported Mild or moderate TBI TSI/D = <2 weeks following hospital discharge	Telephone-based questionnaire administration Rehab-based nurse practitioner One instance of follow-up at 2 weeks following inpatient discharge	Synchronous No reported use of proxy	RPQ	100% of patients were reached, where post-TBI symptoms in at least one domain (emotional, somatic, cognitive) remained present in 100% of cases Referral for additional formal assessment, symptom management, and advice was provided in 37% of cases
Sy et al., 2017 (USA)^57^	Observational (cross sectional and longitudinal analysis as part of wider cohort study)	To evaluate feasibility of a multi-dimensional telephone-administered cognitive test in individuals with moderate-severe tTBI	*n =* 463 (1y) and *n =* 386 (2y) adult TBI patients at home/community Not reported Moderate to severe TBI TSI/D = up to 2 years post-injury	Telephone-based questionnaire administration No defined facilitator Two instances of delivery a year apart	Synchronous No reported use of proxy	Brief Test of Adult Cognition by Telephone (BTACT)	Of the participants independently completing the questionnaire (year 1 = 60%, year 2 = 62%) completion rates ranged from 83% to 88% Of the entire sample, completion rates ranged from 60% to 70% for year 1, and 56% to 64% for year 2. Completion rates lower in participants tested in Spanish (39% to 69%)
Licona et al., 2017 (USA)^76^	Quasi-experimental (test-retest)	To evaluate neuropsychologicalal assessments by telephone on patients with mild-severe TBI to facilitate follow-up evaluations and research studies when in-person assessment is not feasible	*n =* 21 adult (military and veteran) TBI patients at a polytrauma rehabilitation center Average age (range) = 49y (31-71) Sex (F) = 9% All-severity TBI (mild *n =* 7, moderate *n =* 5, severe *n =* 11) TSI/D = within 6 months post-injury	Telephone-based neuropsychological assessment No defined facilitator Two instances several weeks apart (median 15 days, range 7–62 days)	Synchronous No reported use of proxy	Neuropsychological assessment battery (including standard verbally administered tests of attention, working memory, processing speed, language, memory, executive skills, and auditory-verbal adaptions of trail-making)	43% (*n =* 10) completed all 17 tests 91% (*n =* 21) completed 15 tests Telephone testing providing reliable scores across multiple domains even in patients with significant deficits, described as useful for those who find it difficult to travel
**Sutiono et al.^a^, 2017 (Indonesia)^48,79^**	Descriptive	To describe the patients' pathways into RSHS, the pathways following discharge, and the feasibility of following up this patient population by telecommunication	*n =* 178 adult neurosurgical patients (*n =* 104 TBI patients) at home/community *n =* 217: Average age (SD, range) = 41y (14.6, 18–84) Sex (F) = 41% All-severity TSI/D = within 3 months of discharge	Telephone-based follow-up assessment Dedicated neurosurgery nurse Three instances at 1, 2, and 3 months following hospital discharge	Synchronous Use of proxy (designated family member) in some instances to initiate contact with patient	Health-related quality of life (EQ5D5L) at each instance Glasgow Outcome Scale-Extended (GOS-E) at 3 months Technology evaluation: retrospective analysis for feasibility by recording numbers admitted/eligible/consented/able to be followed-up among reasons for loss of contact Anecdotal evaluation of telephonic follow-up experience	Despite difficulty, all patients were able to be reached with no dropouts 55% of patients answered the phone on first contact, whereas 42% required between 2 and 5 attempts before contact was made All but one patient owned regular cell phones. One patient owned a smartphone allowing assessment by videoconference. For 3%, 5+ contact attempts were made Patients were happy to be contacted by telephone due to support provided and opportunity to ask condition-related questions
**Shahrokhi et al., 2018 (Iran)^69^**	Experimental (RCT)	To assess the effect of telenursing on referral rates of patients with head trauma and their family's satisfaction after discharge	*n =* 72 (*n =* 35 intervention, *n =* 33 control following *n =* 4 exclusion) adult TBI patients at home/community *n =* 68: Average age (SD) = 34.11y (12.34)[I], 31.12 (10.83)[C]. Sex (F) = 26.5% Mild to moderate “head injury” (GCS 11–15 at admission) TSI/D = from 12 weeks of discharge	Intervention group: telephone-based caregiver-reported patient status checklist, with telenurse available at any time Telenurse Intervention group: one instance per week for 12 weeks, with caregiver able to contact telenurse as desired Control group: one instance at 12 weeks	Synchronous Full use of proxy; no patients directly communicated with	Generalized patient status checklist for caregiver, including demographics and characteristics, outcomes of care (e.g., readmission, referrals pressure ulcers) Cause of caregiver calls to telenursing also reported Technology evaluation: satisfaction with telenursing service	*n =* 4 excluded (*n =* 1 intervention, *n =* 3 control) for consecutive 3-week non-response or where home nursing services used 53.8% of caregivers satisfied with telenursing program Telenursing program resulted in statistically significant less referrals to physicians (25.7%) versus control group (39.4%)
**Xu et al., 2018 (Uganda)^49^**	Descriptive	To describe the use of phone surveys developed and conducted in the 40 participants’ language to assess mortality, neurological outcomes, and follow-up health care	*n =* 1167 adult and pediatric patients with mixed neurosurgical pathology (TBI, spina bifida, tumor, hydrocephalus, and miscellaneous) at home/community Of those surveyed (*n =* 870), *n =* 740 (85%) with TBI *n =* 870: Median age = 26y Sex (F) = 19% *n =* 596: Median GOS-E = 8 (GR+) Mean GOS-E = 6.83 (GR-) Median TSI/D = 1.53y	Telephone-based survey administration with prospective record electronic database Research assistants in patient's language One instance of follow-up (average duration 20 min)	Synchronous Use of proxy (designated family member) in some instances to initiate contact with patient or collection of demographics	GOS-E or pediatric version (GOSE-peds) General survey items pertaining to: quality of life (continuing physical deficits), activities of daily living (ADLs), ability to speak and follow commands, perform household chores, school and work function, psychosocial function, and subjective return to baseline functional status Additional follow-up health care also assessed	Utilizing telephone, there was a 74.5% response rate (*n =* 870) Of those reached, no patient refused telephone assessment 70% of those who survived pre-discharged (*n =* 1167) had a phone number on file
**Laytin et al., 2018 (Ethiopia)^10^**	Observational (retrospective cohort study)	To assess the feasibility of telephone-administered interviews as a means of collecting follow-up data in this context; to pilot a telephone-administered interview tool for collecting data about long-term functional outcomes after injury; and to collect preliminary data about patients’ long-term functional outcomes after hospital encounters due to injury	*n**** =*** 397 adult mixed trauma patients (*n =* 111 neurological injury) at home/community *n =* 397: Average age (SD) = 32.8y (14.8) Sex (F) = 16.1% Not reported TSI/D** = w**ithin 6 months of discharge	Structured telephone-administered interviews Data clerk One instance of delivery at 6 months post-discharge	Synchronous Use of proxy (“surrogate”: relative, friend, caretaker) where patient unable to respond themselves	Glasgow Outcome Scale Extended (GOS-E)	Over half (*n =* 208) of initially identified patients (*n =* 397) were unable to be reached due to telephone contact details being emergency contacts and/or emergency bystanders, or were believed to not answer due to not recognizing the phone number Formal reasons recorded, where available in *n =* 131 (63%) included: *n =* 92 (72%) not having a valid number on file, *n =* 37 (28%) with a telephone line out of service or not answered on 3 attempts, and *n =* 2 (2%) of contacts made with someone who did not know the current condition of the subject or how to contact them
**Vaca et al., 2018 (Uganda)^19^**	Descriptive	To describe the use of a novel method of telephone surveys to conduct the first-ever long-term follow-up in Uganda to elucidate the outcomes of pediatric head trauma patients treated at the national referral hospital	*n =* 142 pediatric TBI patients and their caregivers at home/community Median age (range) = 6y (0.17–17) Sex (F) = 29% All-severity TBI admission GCS = mild 54%, moderate 31%, severe 15% TSI/D (median) = 1.48y	Structured telephone survey Ugandan research collaborator One instance of follow-up at either 1y or 2y from discharge	Synchronous Full use of proxy (pediatric patient's caregiver)	GOSE-Peds Quality of life (physical and psychosocial deficits, ability to carry out ADLs) Further care sought since discharge, mortality	Average call duration 20 min With up to 5 contact attempts, achieved a 61% response rate (of initial *n =* 232 patients identified), representing 67% of patients receiving treatment and discharge in 12 months with a phone number on file Suggested as a suitable alternative for home visits for a large referral hospital
**Underwood et al., 2019 (Ireland)^50^**	Descriptive	To investigate the prevalence of PCS 1-year post-injury in patients who were treated for mild traumatic brain injury (mTBI) in the CDU of CUH's Emergency Department.	*n =* 57 Adult TBI patients at home/community Median age (range) = 40y (27.5 - 57.5) Sex (F) = 42.1% Mild TBI TSI/D = 1-year post-discharge	Structured telephone assessment No defined facilitator One instance of follow-up at 1 year post-discharge	Synchronous No reported use of proxy	RPQ (SF-12) EQ5D5L	51% response rate (*n =* 57) of initial *n =* 112 attempted after 4 attempts
**Ketchum et al., 2019 (USA)^60^**	Observational (prospective cross-sectional cohort)	To assess the contribution of a brief telephone assessment of cognitive function on prediction of return to work at 1 year following moderate to severe TBI	*n**** =*** 320 adult TBI patients at home/community Age range = 18–64y Sex (F)** =** Not reported Moderate to severe TBI TSI/D** = 1** year following injury	Structured, brief telephone assessment No defined facilitator One instance of assessment at 1-year post-injury	Synchronous No reported use of proxy	BTACT Return to work & employment status	BTACT telephone assessment added significantly to predicting return to work following TBI
**Schlichter et al., 2020 (USA)^52^**	Descriptive (quality improvement initiative in observational cohort format)	To determine the feasibility of measurements of physical function, cognition, and quality of life in patients requiring neurocritical care	*n**** =*** 1324 adult patients with mixed neurological diagnosis (*n =* 218 TBI) at home/community Average age (SD) = 59.5y (17.6) Sex (F) = 45.3% Not reported	Structured telephone assessment utilizing secure web-based data capture platform (REDCap) Clinical nurse, physician, or dedicated research coordinator (all trained in assessment) One instance between 3 and 6 months following discharge	Synchronous Use of proxy (caregiver) for assistance of patient-report or reporting mortality	Modified Telephone Interview for Cognitive Status (mTICS) Patient-reported modified Rankin Scale (mRS) Patient-reported GOS-E Patient-reported EQ5D5L Overall “visual analog scale” health question, on a scale of 0 to 100	Of all neurological diagnosis cohorts, overall loss to follow-up was 23.6% (*n =* 313 of 1324) at a mean (SD) time of 4.4 (0.8) months after initial admission 94% of patients or caregivers who answered calls did so by the second attempt Of remaining TBI patients (*n =* 123), 24.4% did not answer (*n =* 30) On average, completed telephone assessments required 21.9 min to deliver across all diagnosis cohorts
**Rhame et al., 2020 (USA)^53^**	Descriptive (retrospective analysis of quality improvement initiative)	To describe the implementation and utilization of a neurotrauma hotline at a Level 1 trauma center	*n**** =*** 817 unique TBI patients at home/community represented by *n =* 1205 calls to the service No patient demographics reported. Caller demographics (approximate) = patients = 29%, family/friend = 23%, outside provider/staff = 18%, internal provider/staff = 27% Not reported	Neurotrauma telephonic hotline (serviced weekdays, 9am to 5pm) provided to patients upon discharge, with electronic record access for facilitator. Out of hours service provided by voicemail messaging or access to on-call neurosurgeon Registered nurse with TBI expertise *n =* 1205 calls over 12-month period (*n =* 817 unique callers, *n =* 388 repeat callers) with an average of 3.3 calls/day	Synchronous/Asynchronous Proxies able to access service	No reported use of outcome measures administered.	*n =* 1205 calls over 12 month period (*n =* 817 unique callers, *n =* 388 (28.2%) repeat callers) with an average of 3.3 calls/day Calls from patients accounted for approximately 58.9% of system usage Calls were answered live 29.5% of the time. Those not answered live were answered at a median time of 3h 18 min from initial call Highest volume of calls received were in December (*n =* 132) and lowest in February (*n =* 68) Two highest reasons for calls were appointments (36.8%) or to seek advice (32.1%)

^a^ Denotes research published in two parts.

[C], control; CVA, cerebrovascular accident; ED, emergency department; F, female; h, hours; min, minutes; GCS, Glasgow Coma Score; [I], intervention; LoC, loss of consciousness; RCT, random controlled trial; SD, standard deviation; TSI/D, time since injury or diagnosis; w/o, without; y, years.

### SMS-based follow-up

The studies included demonstrated a multi-modal use of SMS-based technology (Table 2). Four studies demonstrated SMS exclusively as the means of data collection, often in an asynchronous and automated manner, through the delivery of timed symptom assessments to patients in the community,^43,56,59,66^ whereby responses were often logged in a database for later review. Two studies, despite not using SMS directly in the collection of outcome data, utilized SMS to deliver prompts or updates. These text messages prompted the patient to log their current status and well-being on other systems such as a secure website or app-based portal.^67^ In another study, SMS was used as a reminder system, informing patients to expect to receive shortly a telephone call, with a focus on improving telephonic response and attrition rates.^45^

**Table 2.** Citations Reporting the Use of SMS-Based Follow-Up

Author, year (Country)	Study design (author definition)	Study aim/Objective	Sample Population demographics TBI characteristics	Follow-up technology (FUT) description Clinical facilitator f Sessions & instances count	Synchronicity Use of proxy	Constructs & outcome measures deployed	Response/Success/Compliance rates
**Smith et al., 2012 (USA)^43^**	Descriptive (pilot)	To assess the utility of mobile health (mHealth) technologies, including personal digital assistant-based ecological momentary assessment and two-way interactive text (SMS) messaging, for providing treatment feedback to clinicians, encouraging and motivating veterans throughout treatment, and monitoring participants for relapse after treatment discharge	*n**** =*** 27 adult (military veterans or active members) traumatic brain injury (TBI) patients with/without PTSD at home/mental health providers : Sex (F)** =** 0% Mild TBI	In follow-up phase: SMS messaging (ecological momentary assessment) between patient and clinical staff/patient's identified “buddies,” and motivational reminder messages Clinical staff, patient “buddies” (relative/family member/friend) Approximately 10 “check-in” prompts per month up to 3 months	Asynchronous No reported use of proxy in response, although “buddy” or clinical staff notified of contact lapse/below-threshold check-in response for 1-1 follow-up.	Generalized Likert scale “check-in” question "How are you doing overall" with (1 = “great” to 5 = “lousy”) and unidirectional motivational messages	91% participants remained engaged (1 response/30 days) at 90 days Average days enrolled in messaging = 72.2 days Average check-in prompts delivered = 9.2 over 30 days Average check-in responses = 8.2 over 30 days
**Suffoletto et al., 2013 (USA)^66^**	Experimental (randomized controlled trial [RCT])	To examine whether patients with mild traumatic brain injury (mTBI) receiving text messaging-based education and behavioral support had fewer and less severe post-concussive symptoms than those not receiving text message support, and to determine the feasibility of using text messaging to assess daily symptoms and provide support to patients with mTBI	*n**** =*** 43 (*n =* 18 intervention, *n =* 25 control) adult TBI patients at home/community Average age (SD) = 30 (9) Sex (F) = 56% Mild TBI TSI/D: convenience sample from emergency department discharge	Timed, SMS-based symptom assessments with symptom-specific education and reassurance Blinded examiner 3 timed questions (9 am, 1 pm, 5 pm) per day over 14 days	Asynchronous No reported use of proxy	Likert scale questions (0 = none to 4 = severe) across three domains (somatic: headaches; cognitive: concentration difficulties; emotional: anxiety or irritability), adapted from the Rivermead Post-concussion Symptoms Questionnaire (RPQ)	84% (*n =* 36) completed 14-day SMS follow-up 93% felt that messaging system was useful to help them self-manage and understand symptoms Over 14 days, 74% completed 9 am headache assessment, 96% completed 1 pm difficulty concentrating assessment, and 97% completed 5 pm irritability/anxiety assessment Among completed assessments, between 49% and 54% completed <1h, and 29% to 54% completed <5 min
**Anthony et al., 2015 (USA)^56^**	Observational (prospective cohort study)	To determine the amount of within-day variation of Concussion Symptom Severity Scores (CSSSs) in athletes with a clinically diagnosed concussion	*n**** =*** 14 youth (sports-related) concussion patients at home/community Age range** =** 14–22y Clinically diagnosed symptomatic concussion (CSSS score 10<)	Automated, timed SMS-based symptom checklist (“text-messaging robot”) with scheduling database No defined facilitator Five scheduled assessments per day for 30 days or until CSSS score of 0 (asymptomatic) for 7 consecutive days	Asynchronous No reported use of proxy	Concussion Symptom Severity Score (CSSS), calculated via SMS-delivered Post Concussion Symptom Score (PCSS)	804 completed surveys (24,180 messages) *n =* 3 subjects had inadequate response rates Average follow-up duration of 23.9 days Time of day did not confound responses
**Pacella et al., 2018 (USA)^59^**	Observational	To examine changes in post concussive symptoms (PCS) over the acute post-injury recovery period, focusing on how daily PCSs differ between mTBI and other injury types	*n**** =*** 108 adult mixed trauma patients (*n =* 39 mTBI, *n =* 16 head injury w/o TBI, *n =* 53 non-head-injured trauma control) at home/community *n =* 39 TBI patients Average age (SD) = 32y (12.1) Sex (F) = 49% Mild TBI TSI/D = from ED discharge	Automated SMS-based self-reported symptom assessment with response storage on electronic database Research team phone Three timed queries per day (9 am, 1 pm, 5 pm) for 14 days	Asynchronous No reported use of proxy	Experience sampling method, using 3 symptom queries with a 5-point Likert scale to mirror the RPQ, assessing: somatic (headaches), cognitive (difficulty concentrating), and emotional (anxiety or irritability) at 9 am, 1 pm and 5 pm, respectively	Of the 14 total queries, average of 11.4 completed for headaches, 11.9 for concentration, and 11.6 for anxiety. Between 88% and 91% of subjects completed each PCS report on at least one day Between 35% and 41% of subjects completed these reports every day Low levels of education were the only variable associated with missing outcome reports (those with less than college education had higher odds of non-completion)

ED, emergency department; F, female; h, hours; min, minutes; PTSD, post-traumatic stress disorder; SD, standard deviation; TSI/D, time since injury or diagnosis; y, years.

### Smartphone application-based follow-up

The second technology most frequently used in the remote collection of outcome data were mobile applications, or “apps,” installed on Apple and Android devices such as smartphones and tablets (Table 3) (*n =* 9).^51,54,58,61,67,72,73,77,78^ Such implementations of mobile applications ranged from gamified symptom journals and social networking^72^ to delivering questionnaires for EMA.^51,58,73^ Three studies described the further use of the device's onboard sensors and additional functionality in the delivery and collection of data, including native “push notifications” to prompt patients to input data,^78^ the GPS tracking function in the collection of activity and community participation data,^61^ or in one study, the Apple iPod Touch's in-built accelerometer to capture objective measurements of physical activity.^54^

**Table 3.** Citations Reporting the Use of Smartphone-Based Folllow-Up

Author, year (country)	Study design (author definition)	Study aim/Objective	Sample Population demographics TBI characteristics	Follow-up technology (FUT) description Clinical facilitator Sessions & instances count	Synchronicity Use of proxy	Constructs & outcome measures deployed	Response/Success/Compliance rates
Juengst et al.^a^, 2015 (USA)^73^	Quasi-experimental (pilot study of prospective repeated measures design)	To assess pilot feasibility and validity of a mobile health (mHealth) system for tracking mood-related symptoms after traumatic brain injury (TBI)	*n**** =*** 20 adult TBI patients at home/community Average age (SD) = 36.7y (12.4) Sex (F) = 40% All-severity TBI, classed as initial GCS <12 or 13–15 with positive neuroradiological findings consistent with TBI TSI/D (SD) = 5.2y (3.6)	Patient-facing smartphone application-based ecological momentary assessment (EMA), Personalized EMA Rehabilomics Forms for Rehabilitation Medicine (“iPerform”) and clinician-facing web-based portal (iPerform Portal). App has additional communication functions allowing patients to send text messages and clinicians/researchers to send emails to patients For comparison, traditional telephone-based interview Clinician/Researcher team One assessment per day during patient-identified preferred 3-h window. Two-week schedule of varying assessments. Schedule repeated 4 times for 8 weeks total	Asynchronous No reported use of proxy	Daily: Patient Health Questionnaire 2 (PHQ-2) Generalized Anxiety Disorder 2 (GAD-2) General fatigue statement agreement using 7-point Likert scale (1 = strongly disagree, 7 = strongly agree) Positive and Negative Affect Schedule (PANAS) Biweekly: Patient Health Questionnaire 9 (PHQ-9) Generalized Anxiety Disorder 7 (GAD-7) PANAS Technology evaluation: Compliance (retrospectively calculated) Satisfaction: six 7-point Likert scale questions assessing usability and satisfaction completed bi-weekly via telephone Usability: Telehealth Usability Questionnaire (TUQ) conducted during final week of assessment	73.4% assessments completed as scheduled; 79.8% completed as a whole 6.3/7 (SD 0.8) patient satisfaction with iPerform smartphone application assessment. From the TUQ, 6.2/7 (SD 0.8) reported ease of use, 4.3/7 (SD 1.7) for reliability and 5.5/7 (SD 1.1) for satisfaction with iPerform Low reliability thought to be due to technical problems faced by participants throughout study (notification receipt errors or application crashing and/or not submitting assessment) High correlations with standard telephone-interview supporting validity of smartphone-based mood-related EMA in this population
Pavliscsak et al., 2016 (USA)^67^	Experimental (secondary analysis of multi-site prospective random controlled trial [RCT])	To examine engagement with a mobile application (“mCare”) for wounded service members rehabilitating in their communities. Many had behavioral health problems, TBI), and/or post-traumatic stress disorder (PTS), and to examine associations between service members’ background characteristics and their engagement with mCare	*n**** =*** 95 adult (military service members) of mixed diagnoses with behavioral health, PTSD, and/or TBI at home/community *n =* 95: Average age range (SD) = 34.7y (10.3) to 39.7 (10.4) Sex (F) = 0% to 27.3% Not reported	Bi-directional mobile health smartphone application “mCare” utilizing SMS updates/prompts, and secure encrypted website to deploy scheduled app-based status questionnaires Care team members Daily questionnaires (seven varieties delivered once/week) at 10 am local time for up to 36 weeks	Asynchronous No reported use of proxy	Questionnaires included: general status, pain status, energy and sleep status, anger management, relationship status, transition goal status, mood status Weight status sent once per month	Participants usually responded to 60% of the questionnaires weekly, generally in 10 h; however, participants with behavioral health problems had several weeks with <50% response/longest response times. Older age and higher general well-being schedule scores were associated with greater and faster responses
Wiebe et al., 2016 (USA)^54^	Descriptive	To determine the feasibility of EMA following youth concussion, gather real-time reports of cognitive and physical activity, and compare objective measures with real-time reported symptoms among youth during recovery after concussion	*n**** =*** 34 pediatric concussion patients at home/community Median age (range) = 15y (13–16) Sex (F)** =** 47% Concussion, diagnosis based on Zurich consensus diagnostic criteria Median TSI/D (range)** =** 9 days (5–13)	Apple iPod Touch with custom application and use of in-built accelerometer for administration of questionnaires following randomly timed prompts by EMA No defined facilitator Two weeks of daily symptom reports and physical activity monitoring	Asynchronous No reported use of proxy	Post-Concussion Symptom Scale (PCSS) Activity questionnaire of daily activities Step count Daily cognitive rest and exertion (by calculating composite score of measurements in number of texts sent, min of screen time and gaming, and min of reading or schoolwork)	*n =* 28 (82%) responded to more than 80% of symptom questionnaire prompts *n =* 34 were enrolled for a median of 6 days after injury (range 3–10)
**Worthen-Chaudhari et al., 2017 (USA)^72^**	Quasi-experimental (two-phase, non-randomized, open label design)	To evaluate whether the app would be feasible for use by youth with unresolved concussion symptoms as a complement to standard medical care (Phase 1), and to assess whether recovery profiles differed between youth who augmented medical care with the app and those who received medical care alone (Phase 2)	*n**** =*** 42 pediatric concussion patients at home/community (Phase 1 *n =* 20, Phase 2 *n =* 19) Phase 1: Average age (SD, range) = 15.6y (1.6, 13–18) Sex (F) = 70% Phase 2: Average age (SD, range) = 15.6 (1.7, 13–18) Sex (F) = 77% Physician-diagnosed concussion or mild TBI (SCAT-3 score 4<) TSI/D = 3 weeks to 12 months post-injury	Smartphone application “SuperBetter” encompassing a gamification-based symptoms journal, “Battle Royal Power Pack,” with personal social networking for in-app activity monitoring Research coordinator and friends/family One logged activity per day for 5 days over 3 weeks (target dose of 15 logged activities)	Asynchronous No reported use of proxy	Phase 2: Concussion symptom severity on the SCAT-3 checklist score. Secondary: optimism (measured by Life Orientation Test-Revised [LOTR]), depression (measured by Center for Epidemiological Studies - Depression Child [CES-DC]) Technology evaluation: Phase 1: Number of participants completing the intervention relative to all enrolled. Application use (%Play) expressed as % of target dose in first 3 weeks of intervention Satisfaction with intervention (7-point Likert, 1 = high, 7 = low)	In Phase 1, *n =* 14 (70%) completed the intervention Of the 14 participants in phase, high satisfaction (median *n =* 2, range 0) was reported Application use was high in both phases (Phase 1: median %Play = 110% ± 22% of target dose, Phase 2 median 113% ± 8%). In Phase 1, barriers to compliance (from remaining *n =* 6) included discontinuation of medical care (*n =* 3), unanticipated difficulty with home Internet access (*n =* 1), concomitant illness (*n =* 1), and competing extracurricular schedules (*n =* 1) Symptoms and optimism improved more for the experimental than for the active control cohort
**Juengst et al., 2017 (USA)^58^**	Observational (prospective cohort)	To investigate the within- and between-person variability in self-reported emotional symptoms and fatigue, measured through EMA, among individuals with chronic TBI.	*n**** =*** 21 adult TBI patients at home/community Not reported Chronic TBI TSI/D** =** 6< months post-injury	Smartphone-based EMA on Apple and Android smartphones or tablets No defined facilitator Daily ecological momentary assessment for 8 weeks	Asynchronous No reported use of proxy	Alternating assessments between: affect, assessed by Positive and Negative Affect Schedule (PANAS) Mood, assessed by GAD-2 and (PHQ-2 General fatigue item using 7-point Likert (“Fatigue interferes with my work, family or social life”)	Of *n =* 21 consented, *n =* 17 (81%) completed any daily assessments in the 8-week period EMA demonstrated statistically significant fluctuations in affect and mood domains, demonstrating applicability of EMA in chronic TBI cohorts to adequately capture temporal symptoms over time
**Graham et al., 2017 (USA)^77^**	Quasi-experimental (prospective, repeated measures design)	To assess the feasibility of using smartphone application technology to assess participation following TBI	*n**** =*** 10 adult TBI patients at home/community Working-age adults Not reported	Smartphone application-based EMA No defined facilitator 4 times per day, daily for 4 weeks	Asynchronous No reported use of proxy	Mobile Participation Assessment Tool (mPAT) Technology evaluation: compliance, smartphone application ease of use, comfort using smartphone application to answer questions	82.9% of all scheduled assessments were completed Compliance varied by week (80.4%-90.6%) and time of day (79.4%-84.61%) On average, on a scale of 1-5, patients reported mPAT was easy to use (mea*n =* 4.5 SD 0.71), was an acceptable way to measure their participation (mea*n =* 4.3 SD 1.06), and they were satisfied with the mPAT as a measure of their participation (mea*n =* 4.2 SD 1.03). Additionally, participants reported feeling comfortable using the application (mea*n =* 4.6 SD 0.52) and that it is an acceptable way to answer questions (mea*n =* 4.6 SD 0.52)
Sufrinko et al., 2019 (USA)^78^	Quasi-experimental (prospective repeated measures)	To evaluate mobile ecological momentary assessment (mEMA) as an approach to measure sport-related concussion (SRC) symptoms, explore the relationships between clinical outcomes and mEMA, and determine whether mEMA was advantageous for predicting recovery outcomes compared to traditional symptom report	*n =* 20 pediatric/adolescent concussion patients at home/community Average age (SD, range) = 15.35 (1.98, 12–19) Sex (F)** =** 40% Diagnosed with isolated sports-related concussion TSI/D** =** within 72 h of injury	Specialized custom smartphone application (iOS and Android) forEMA, “mEMA,” with incorporated prompts (push notification) No defined facilitator Three instances of assessment at predetermined fixed time blocks (morning, afternoon, evening) daily until second follow-up or medical clearance (whichever first)	Asynchronous No reported use of proxy	Neurocognitive testing by Immediate Post-Concussion Assessment and Cognitive Testing battery (ImPACT) with PCSS embedded within Vestibular Ocular Motor Screening (VOMS)	90% of participants responded to mEMA prompts with an overall response rate of 52.4% (*n =* 1155 prompts) Average response rate of 50.4% (SD 29.3) per participant responded to throughout the study, with a range of 5.4% to 95.2% Average prompts received *n =* 64, range 19–173) There was no correlation between number of prompts received and the response rate (Spearman rho = 0.08, *p* = 0.77) Participants were less likely to respond as days since injury increase (OR = .91, 95% CI: 0.87-0.94, *p* < 0.001) Response rate differed by age with older participants less likely to respond (OR = 0.56, 95% CI: 0.34-0.93, *p* = 0.026). There was no association between response rate and time of day (morning = 50.1%, afternoo*n =* 52.9%, evening = 49.8%; *p* = 0.411) There was no difference in response rate for initial symptom burden (OR = 0.97, 95% CI: 0.92-1.03; *p* = 0.354)
**Juengst et al.^b^, 2019 (USA)^51^**	Descriptive (secondary analysis of prospective descriptive pilot study)	To investigate within-person variability in daily self-reported emotional and fatigue symptoms and factors associated with high within-person variability among individuals with chronic TBI	*n**** =*** 18 adult chronic TBI patients at home/community Average age (SD) = 38.3y (12.7) Sex (F) = 72% All-severity TBI TSI/D** =** 12y (67%), range 2–27 years post-injury	Smartphone-based l EMA on Apple and Android smartphones or tablets No defined facilitator Daily instance of assessment for 8 weeks (56 time-points)	Asynchronous No reported use of proxy	At odd-numbered time-points: PHQ-2, GAD-2, and general 7-point Likert scale question regarding impact of fatigue on daily life On even-numbered time points: PANAS	Not reported
**Wen et al., 2021 (USA)^61^**	Observational (parallel observational cohort study)	To evaluate the feasibility of a smartphone application (app) called MOVES to objectively measure community participation; and compare MOVES with a self-report questionnaire, and differences between veterans with mTBI and civilians without TBI	*n**** =*** 16 (*n =* 11 veterans with TBI, *n =* 5 civilians with no TBI) at home/community Average age (SD) = 36.14y (4.9) [veteran cohort], 33.00 (4.9) [civilian cohort] Sex (F) = 0% [both cohorts] Mild TBI TSI/D** =** not reported	Smartphone application (MOVES) for iOS and Android, utilizing inbuilt phone GPS tracking. Store-and-forward of data by secure messaging email through a secured portal (MyHealtheVet) Research team Daily activity/GPS tracking for up to 6 weeks	Asynchronous No reported use of proxy`	GPS-based activity and location monitoring (MOVES Storylines) Technology evaluation: satisfaction questionnaire at week 6 (ten 5-point Likert questions) Daily documentation of unexpected events (technology-related) Perceived accuracy of MOVES Storylines (Perceived Accuracy Daily Logs)	There was a 75% retention rate (*n =* 11) Participants reported an average of 90% accuracy between the MOVES Storylines and a self-reported questionnaire, Participation Assessment with Recombined Tools Objective (PART-O) Overall, all participants reported they were mostly satisfied (3.65/5) with a range of 2.1 to 4.8 The lowest satisfaction rating was received for the effort required with sending the application data to the research team with an average of 2.68/5 between veteran and civilian cohorts The highest satisfaction rating for veterans was an item reporting the effort required to take the smartphone on their persons during the day with an average of 4.29/5 The highest satisfaction rating for civilians was an item reporting the ability of the MOVES application to capture their activities in the community with an average of 4.4/5 It took between 10 to 15 min to score each daily story line per person, with an estimated 4 to 5 h required for 28 days of data per person

b51 This was a secondary analysis of a previous pilot feasibility study, ^a73^, and is included as a separate entry for totality.

CI, confidence interval; F, female; h, hours; min, minutes; OR, odds ratio; SD, standard deviation; TSI/D, time since injury or diagnosis; y, years.

### Videoconferencing-based follow-up

Three studies utilized videoconferencing exclusively for remote assessment (Table 4).^70,71,75^ Two studies, by the same authorship group, described the use by the assessing speech-language pathologist of two remote-controlled robotic web cameras.^70,71^ Additionally, in their second article with a system re-design, the authors describe concurrent automatic store-and-forward facilities integrated into their system, enabling video and audio data of higher quality than that streamed over the 128kbit/sec videoconference connection to be sent to the assessing clinician for later review.^71^ Further, one study reported the use of a novel, custom, portable home care activity desk (HCAD) installed in the patient's home. Each unit consisted of sensorized tools and videoconferencing facilities, providing store-and-forward capability between the patient's home and hospital servers.^65^ One study additionally described the use of telephone as a backup option utilized in cases of videoconferencing technical difficulty.^75^

**Table 4.** Citations Reporting the Use of Videoconference-Based Follow-Up

Author, year (country)	Study design (author definition)	Study aim/objective	Sample Population demographics TBI characteristics	Follow-up technology (FUT) description Clinical facilitator Sessions & instances count	Synchronicity Use of proxy	Constructs & outcome measures deployed	Response/Success/Compliance rates
Huijgen et al., 2008 (Italy, Spain, Belgium)^65^	Experimental (randomized multi-center trial)	To investigate the feasibility of a telerehabilitation intervention for arm/hand function (the Home Care Activity Desk [HCAD] training) in a home setting	Traumatic brain injury (TBI) cohort: *n =* 30 (*n =* 20 intervention, *n =* 10 control) adult TBI patients at home/community Average age (SD)** =** 32 (13)[I], 38 (17)[C] Sex (F) = 20% [I], 30% [C] TSI/D (SD)** =** 7.5y (4.4) [I], 7.8y (2.9) [C]	HCAD consisting of sensorized tools, videoconferencing facilities (2 x webcams) and remote data upload to hospital. Data reviewed in weekly patient-therapist videoconferencing Therapist 30-min sessions per day for 5 days per week (20 days total)	Synchronous/Asynchronous No reported use of proxy	Action Research Arm Test (ARAT) Nine Hole Peg Test (NHPT) Technology evaluation: General user satisfaction (acceptance, aesthetic, ease of use, task difficulty, task appropriateness, general impression of HCAD)	Average usage across pathologies (TBI, MS, stroke) = 30 min per day for 19 days (treatment time 9.5 h/month). Average treatment time similar to usual care Overall compliance varied from 7 to 38 days (recommended 30 min/5 days per week totaling 20 days) As a majority, both patients and therapists satisfied with HCAD. Only aesthetic aspects of system and task difficulty resulted in slightly less satisfaction as a whole
Hill et al.^a^, 2009 (Australia)^70^	Experimental (randomized controlled trial [RCT])	To determine if valid and reliable assessment of apraxia of speech using a standardized assessment tool was feasible via an Internet-based telerehabilitation system	*n**** =*** 11 adult patients with mixed diagnosis (*n =* 2 TBI, *n =* 9 CVA) at hospital research laboratory 15 km from assessor TBI cohort, *n =* 2: Average age = 20y Sex (F) = 50% Not reported TSI/D (TBI cohort, *n =* 2) = 6.5 months average post-injury	Custom real-time videoconference-based assessment using two web cameras mounted on robotic arm over 128 kbit/sec connection. Participant wore headset microphone and earphones. System incorporated concurrent automatic store-and-forward facilities integrated into software for high-resolution video and audio data sharing Speech-language pathologist Single instance of assessment	Synchronous/Asynchronous No reported use of proxy	Apraxia Battery for Adults 2 (ABA-2) Technology evaluation: Participant Satisfaction Questionnaire, consisting of eight items employing 5-point Likert scale	Across diagnosis cohorts, no significant differences were found between the telerehabilitation assessment versus in-person assessments, with moderate to very good agreement indicated All participants eligible to complete the satisfaction questionnaire (*n =* 5) described the audio quality as good or excellent; *n =* 3 described the video quality as good or excellent; *n =* 2 adequate. *n =* 3 described the comfort level during the videoconference sessions as comfortable or very happy, with *n =* 1 having no feeling either way and *n =* 1 described it as uneasy With regards to overall satisfaction, all were satisfied, with *n =* 3 describing the videoconference assessment as more than or very satisfied All but one participant indicated they would be equally satisfied with services being delivered via videoconference versus in-person
Hill et al.^b^, 2009 (Australia)^71^	Experimental (RCT)	To refine the telerehabilitation system used in the Hill et al.^70^ study and re-evaluate this new system with a modified research design to determine validity and reliability of the assessment of acquired dysarthria in adults	*n**** =*** 24 adult patients with mixed diagnosis (*n =* 11 TBI) at hospital research laboratory 15 km from assessor *n =* 24: Average age (range) = 50.2y (16–78) Sex (F) = 37.5% Not reported TSI/D (range) (*n =* 24) = 42.6 month average (6 months to 11y)	Custom real-time videoconference-based assessment using two web cameras mounted on robotic arm over 128 kbit/sec connection. Participant wore headset microphone and earphones. System incorporated concurrent automatic store-and-forward facilities integrated into software for high-resolution video and audio data sharing. Additional data-sharing capabilities that allowed instructional images and videos to be displayed locally versus transmitted allowing more streamlined and efficient assessment Speech-language pathologist Single instance of assessment	Synchronous / Asynchronous No reported use of proxy	Assessment battery including: Informal oromotor assessment Informal perpetual speech assessment Assessment of Intelligibility of Dysarthric Speech (ASSIDS) Technology evaluation: Participant Satisfaction Questionnaire, consisting of eight-items employing 5-point Likert scale	Good strength of agreement was found between the FTF and telerehabilitation assessment methods. The majority of participants (*n =* 10 of *n =* 11) rated both the audio and visual quality as good or excellent All (*n =* 11) participants were comfortable or very happy with the telerehabilitation assessment session, and all participants rated their overall satisfaction as more than satisfied or very satisfied All (*n =* 11) participants reported being confident with the results gained via telerehabilitation assessment, and all were willing to participate in future telerehabilitation assessments The majority of the participants (*n =* 8) stated that they would be equally satisfied with speech pathology services delivered via telerehabilitation methods Only *n =* 4 thought it would be more convenient for them to access speech pathology services in this manner. Of the *n =* 7, *n =* 5 felt that telerehabilitation would not be more convenient for them, and *n =* 2 stated that it was not applicable as they did not have access to the Internet at home
Rietdijk et al., 2017 (Australia)^75^	Quasi-experimental (repeated measures design with randomized order)	To compare in-person with videoconferencing administration of a communication questionnaire for people with traumatic brain injury (TBI) and their close others.	*n**** =*** 20 Adult TBI patients and their close others at home/community Sex (F)** =** 20% “Close others”** =** *n =* 8 parents, *n =* 6 partners, *n =* 3 other family members, *n =* 3 friend of TBI patient Severe TBI (post-traumatic amnesia duration 21–180 days). TSI/D (range)** =** 6< months (8 months to 22y)	Videoconference -based (Skype) assessment with telephone fallback, with in-person comparison conducted in patient's home Clinician researcher One instance of remote assessment paired with one instance in-person between 1 and 2 weeks apart	Synchronous Use of proxy, ‘close others’	La Trobe Communication Questionnaire (LCQ) for patient self-report (LCQ Form S) and their close other (LCQ Form O)	*n =* 1 excluded due to lack of comprehension of questionnaire in either in-person or videoconference setting Of remainder, 89.5% (*n =* 17) received successful administration of videoconference outcome measure Due to connection and quality difficulties, *n =* 2 were administered by telephone No significant differences between videoconferencing and in-person for LCQ score or administration time

a,b Studies conducted by the same authors; ^b^study included separately due to revised methods and a novel patient cohort.

[C], control; CVA, cerebrovascular accident; F, female; h, hours; [I], intervention; min, minutes; MS, multiple sclerosis; SD, standard deviation; TSI/D, time since injury or diagnosis; y, years.

### Miscellaneous technologies for follow-up

Two studies, pooled together as “miscellaneous” (Table 5), described the use of portable touchscreen electronic devices, one proprietary device (PsyMate)^62^ and one commercially available PDA device.^63^ Both studies described auditory prompt capabilities with their devices, enabling semi-randomly scheduled EMA throughout the day.^62,63^ One study previously noted described allowing the patient to choose between a secure Internet website portal or telephonic IVR to submit self-reported outcome data.^55^

**Table 5.** Citations Reporting the Use of Miscellaneous Technology-Based Follow-Up

Author, year (country)	Study design (author definition)	Study aim/Objective	Sample Population demographics TBI characteristics	Follow-up technology (FUT) description Clinical facilitator Sessions & instances count	Synchronicity Use of proxy	Constructs & outcome measures deployed	Response/Success/Compliance rates
**Lewandowski et al., 2009 (USA)^63^**	Observational	To examine the feasibility of a momentary data-gathering method, as well as the sensitivity of the assessment to the subtle and dynamic changes in symptoms of concussion	*n**** =*** 3 pediatric concussion patients and *n =* 3 healthy pediatric individuals at home/community Total: Age range = 14–17y Sex (F) [concussion patients, *n =* 3]** =** 66.6% Symptomatic concussion/mild traumatic brain injury (mTBI) TSI/D** =** average 117 days (range 78–165)	Stylus-based touchscreen personal digital assistant (Palm Pilot 100) based ecological momentary assessment with auditory prompts No defined facilitator Five daily instances of delivery (between 9 and 10 am, 11 am and 12 pm, 2 and 3 pm, 5 and 6 pm, 8 and 9 pm) for 5 consecutive weekdays. Prompts received every 5 min until response received	Asynchronous No reported use of proxy	Setting context Symptom Severity Scale (SSS), consisting of 13 symptom items and 7-point Likert scale Functional Status Scale (FSS), consisting of 15 functional impairment items and 7-point Likert scale	Each assessment took approximately 3–5 min There were no technical difficulties reported Of a possible 75 assessments, 70 were complete, demonstrating a compliance rate of 93.3% The device was reported to be not disruptive to others in a school setting or to the students using it
**Lenaert et al., 2019 (Netherlands)^62^**	Observational (longitudinal observational study)	To investigate the feasibility of using experience sampling method (ESM) in individuals with acquired brain injury (ABI), to explore the usability of ESM data on a clinical level, by illustrating the interactions between person, environment, and affect	*n**** =*** 17 adult ABI patients (*n =* 8 traumatic brain injury [TBI]) at home/community *n =* 17: Average age (SD, range) = 44.2y (14.5, 18–65) Sex (F) = 53% Not reported	Touchscreen electronic device, “PsyMate,” with semi-randomly scheduled auditory prompts for ecological momentary assessment No defined facilitator 10 instances delivered daily at semi-random scheduled times over 6 days	Asynchronous No reported use of proxy	Positive & Negative Affect Schedule (PANAS) Location and social context including appraisal using bipolar scale Activities and physical well-being (including fatigue), including appraisal using bipolar scale Technology evaluation: subjective experiences assessed by two debriefing questionnaires (18, 7-point Likert items in total) on user friendliness and general acceptability of methodology	Average response rate of 71.18% (*n =* 726) following delivery of 1020 prompts 98.76% of prompted reports were completed, with an average of 42.7 questionnaires answered (range 28–57) There were no dropouts reported, with the method experienced as user-friendly The device was reported to have little influence on their activities or social contacts (average = 2.00/7, SD 1.16). There were little to no difficulties reported when using the device (average = 1.77/7, SD 1.36), with the device not experienced as burdensome (average = 2.08/7, SD 1.19) The amount of beeps were not seen as much of a burden (average = 2.08/7, SD 1.19)

F, female; SD, standard deviation.

### Clinical facilitators

Clinical facilitators were most often explicitly described in the article as the research staff (i.e., research coordinators and care managers, research assistants, and clinical researchers)^19,42,49,52,59,61,72,73,75^ or hospital outcome management staff^55^ and blinded examiners.^66^ Nursing personnel^41,52,69^ formed the next largest pool of facilitators, and included specialist psychiatric nurses,^64^ rehab-based nurse practitioners,^47^ or neurosurgical nurse personnel experienced in TBI and/or neurotrauma outcome measure administration.^48,53,80^ Undefined clinical staff,^43^ speech-language pathologists,^70,71^ physicians,^52^ general therapists,^65^ occupational therapists,^68^ and care team members^67^ were described as facilitators in a smaller subset of studies. In three studies, facilitators were primarily external non-clinical staff without familiarity of TBI, such as data clerks,^10^ call center personnel,^44^ or interviewers otherwise undefined.^74^ However, 15 studies did not explicitly report on who facilitated the follow-up reported in the article.^45,46,50,51,54,56–58,60,62,63,76–78,81^ Although it may be assumed that the facilitators of technology in these studies were the author teams themselves, this cannot be confirmed.

Despite not being described as active facilitators, it is of note that in two studies, family members, friends, and relative facilitators were incorporated in the delivery of the follow-up technology,^43,72^ and instead may be designated as “passive facilitators.” One study with a pediatric population describes a “social networking” function built in to the smartphone application, allowing friends and family to connect with and receive notifications of the patient's activities and progress.^72^ An additional study describes how designated friends or relatives could opt in to receive notifications should their injured relative fail to maintain contact with the FUT services, or if they returned a score below a pre-set threshold that warranted further one-to-one contact outside of the FUT.^43^

### Timing of follow-up and time since injury, diagnosis, or discharge

The timing of remote follow-up delivery with respect to the patient's time since injury/diagnosis (TSI/D), or time since hospital discharge, could be established directly or approximated in 77.5% (*n =* 31) of articles retrieved. Studies were broadly grouped together by respective timeframes of: less than 1 month, 1–3 months, 3–6 months, 6–12 months, and 12 months or more. Eight (20%) studies reported remote assessment within a month of discharge or injury;^42,45,47,54,59,66,68,78^ within this group, five studies^54,59,66,68,78^ described recruitment and assessment of participants from discharge up to 2 weeks post-discharge for the assessment of concussion or mild TBI. Three (7.5%) studies^41,48,64^ assessed patients between 1- and 3-months post-injury or discharge, and all utilized telephone as the modality of choice. Five (12.5%) studies^10,52,63,69,76^ conducted remote assessment beyond 3 months and within 6 months of discharge or injury. Four studies (10%) described assessment between 6 and 12 months.^46,58,70,72^ Lastly, 27.5% (*n =* 11) of studies^19,49–51,57,60,65,71,73–75^ depicted an average time-point of remote assessment of 1 year and beyond hospital discharge or injury. Nine studies (22.5%) did not formally define the time-point at which remote assessment was attempted.^43,44,53,55,56,61,62,67,77^

### Intervals between sessions

The most frequently reported (*n =* 14) timeframe of outcome data collection by FUT was at one single time-point following injury or discharge.^10,19,44,46,47,49,50,52,55,60,70,71,74,75^ Of these studies, 11 (*n =* 11, 78.6%) utilized telephone-based technology.^10,19,44,46,47,49,50,52,55,60,74^ One study used two modalities in a single instance of follow-up, namely a secure web-portal and telephone-based IVR system.^55^ Three studies used videoconferencing in a single instance.^70,71,75^

The remaining studies (*n =* 26, 65%) reported more than one outcome data collection point. One study reported follow-up at yearly intervals for 2 years post-injury.^57^ Another study reported two follow-up points at quarterly intervals of 3- and 6-months post-injury.^41^ One study described data collection on a monthly basis up to 3 months following discharge.^48^ Similarly, a further study described fortnightly instances of follow-up by telephone over the course of 4 weeks.^45^ Three studies reported collecting outcomes on a weekly basis^43,64,69^—two of which were by telephone^64,69^—and the first use of SMS is seen at this weekly interval.^43^ In those studies using follow-up technology on a daily basis, eight studies utilized smartphone applications^51,54,58,61,67,72,73^ and one employed a videoconference-based, sensorized HCAD previously described.^65^

**FIG. 3.**
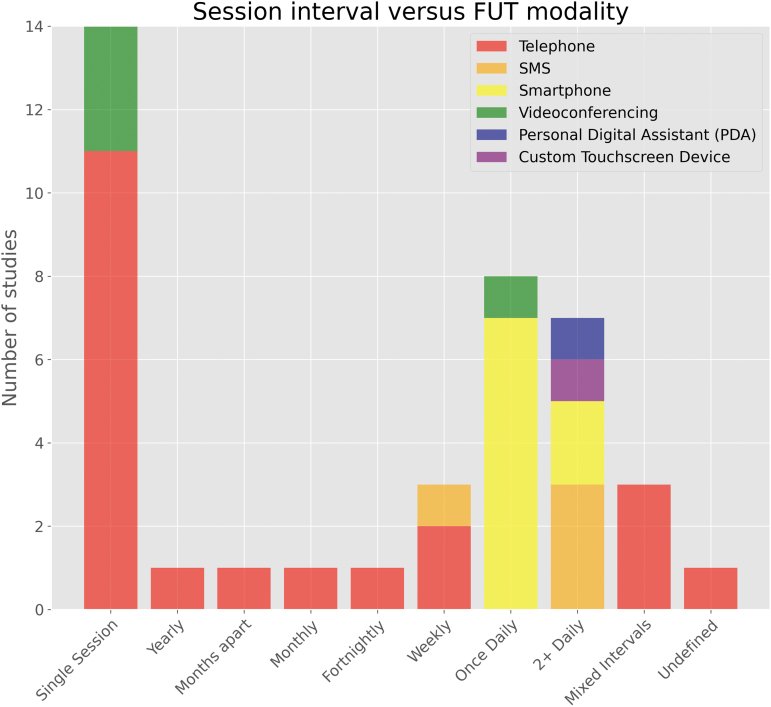
Intervals between remote follow-up sessions with respect to follow-up technology modality. SMS, short message service.

Seven studies reported collecting data multiple times throughout the day^56,59,62,63,66,77,78^; this session interval also used the most diverse range of technologies of the intervals described thus far. Three studies explored the use of SMS up to five times daily,^56,59,66^ and two studies demonstrated the use of smartphone applications up to four times daily.^77,78^ The remaining studies used a PDA^63^ and custom touchscreen device^62^ to examine outcomes up to 10 times daily. Lastly, three studies employing telephone as an FUT collected data at steadily increasing intervals up to 9 months post-injury.^42,68,76^ One additional study, owing to the nature of the telephone-based service (a neurotrauma hotline), was not able to define an interval between sessions; however, it reported an average of 3.3 calls per day over a 12-month period.^53^ A visual summary of the intervals between FUT sessions with respect to technology modality can be found in Figure 3.

### Synchronicity

Half of the studies utilized synchronous or real-time assessment in the collection of outcome data (*n =* 20, 50%).^10,19,41,42,44–50,52,57,60,64,68,69,74–76^ Sixteen (40%) used an asynchronous or store-and-forward approach.^43,51,54–56,58,59,61–63,66,67,72,73,77,78^ Four studies (10%) demonstrated the use of mixed synchronicity,^53,65,70,71^ often utilizing different capabilities of the technology in its service delivery, such as voicemail (asynchronous) services built in to a neurotrauma hotline (synchronous).^53^

### Use of proxies

In 29 studies, there was no reported use of proxies (72.5%).^41,43–47,50,51,54–63,65–67,70–74,76–78^ Eight studies (20%) were extended to both patients or their proxies in remote follow-up.^10,42,48,49,52,53,64,75^ Of these studies, two (5%) used a mixture of both patient and proxy data, usually collected in the same sitting, to assess the patient's well-being.^42,75^ A further two studies described the assistive role of proxies where the patient was unable to directly report their well-being themselves.^10,52^

Two studies (5%) described the role of proxies in assisting initiating contact with the patient if they were initially unable to be reached, although did not further disclose whether the proxies were used to seek information on behalf of the patient.^48,49^ One study, in addition to assisting the patient to report their well-being, used proxies to report data pertaining to mortality as necessary.^52^ One study,^43^ despite not using a proxy to ascertain data regarding the status of health of the patient on their behalf, utilized a clinical staff or “buddy” contact system of friends and family to facilitate further check-in with the patient, should they not respond to the asynchronous technology-based well-being report, or not meet a threshold score indicating they were otherwise well.

Three studies (7.5%)^53,64,69^ described telephone-based support made available for patients and their proxies,^53,64^ and their proxies exclusively,^69^ to share symptoms or well-being status (on behalf of the patient where required), or to seek support at their own convenience, in between scheduled requests or prompts for patient status reports.^64,69^

Three studies (7.5%) described the use of proxies exclusively to assess patients in their FUT deployment, without directly communicating with the patients themselves.^19,68,69^ Pediatric patient cohorts formed the basis of two of these studies,^19,68^ whereby follow-up and outcome data collection was solely provided by the patient's caregivers owing to the patient's age.

### Deployment of outcome measures

Several studies utilized one or more validated outcome measures in their technologies, whereas a small number of studies used internally developed measures, scores, and scales in the remote assessment of patients. The Glasgow Outcome Scale-Extended (GOS-E) was the most frequently used in the studies included (*n =* 6), followed by the Rivermead Post-Concussion Questionnaire or its derivatives (*n =* 5), and the 5-Level EuroQol 5-Dimension instrument (*n =* 4). A full list of outcome measures deployed in FUTs for TBI, ordered by frequency, can be found in Table 6.

**Table 6.** Outcome Measures Deployed via Follow-Up Technologies in TBI Populations

Outcome measure (or derivative)	Abbreviated outcome measure	Number of implementations	Citations
Glasgow Outcome Scale-Extended Glasgow Outcome Scale Extended: Pediatrics (GOSE-Peds)	GOS-E GOSE-Peds	5 2	10,48,49,52,79 19,49
Rivermead Post-Concussion Questionnaire Short-form derivatives assessing key domain- symptom pairs (somatic: headaches; cognitive: concentration difficulty; emotional: anxiety or irritability)	RPQ -	3 2	46,47,50 59,66
5-Level EuroQol 5-Dimension	EQ-5D-5L	4	46,48,50,52
Generalized Anxiety Disorder Assessment Generalized Anxiety Disorder Assessment 2-item	GAD-7 GAD-2	1 3	73 51,58,73
Public Health Questionnaire Public Health Questionnaire 2-item Public Health Questionnaire 9-item	PHQ PHQ-2 PHQ-9	0 3 1	- 51,58,73 73
Positive and Negative Affect Schedule	PANAS	4	51,58,62,73
Post-Concussion Symptom Score Concussion Symptom Severity Score Immediate Post-Concussion Assessment and Cognitive Testing Battery	PCSS CSSS ImPACT	1 1 1	54 56 78
Brief Test of Adult Cognition by Telephone	BTACT	2	57,60
Glasgow Outcome Scale Glasgow Outcome Scale: Hindi variation	GOS GOS: Hindi	0 1	- 44
Medical Outcomes Study Short Form 12-Item Short Form Survey	SF-36 SF-12	0 2	- 46,50
Telephone Interview for Cognitive Status Modified Telephone Interview for Cognitive Status	TICS TICSm	1 1	41 52
Action Research Arm Test	ARAT	1	65
Acute Concussion Evaluation	ACE	1	68
Apraxia Battery for Adults-2	ABA-2	1	70
Assessment of Intelligibility of Dysarthric Speech	ASSIDS	1	71
BSF/A: Functional Independence Measure	FIM	1	41
Center for Epidemiological Studies Depression Scale for Children	CES-DC	1	72
Community Participation Indicators Computer Adaptive Testing Community Participation Indicators	CPI CAT-CPI	0 1	- 55
Functional Status Scale	FSS	1	63
La Trobe Communication Questionnaire, self-reported (Form S) and proxy-reported (Form O)	LCQ	1	75
Mobile Participation Assessment Tool	mPAT	1	77
Modified Rankin Scale	mRS	1	52
Neurobehavioral Rating Scale	NRS	1	41
Nine Hole Peg Test	NHPT	1	65
Ohio State University TBI Identification Method	OSU TBI-ID	1	74
Post-Concussion Symptom Inventory: Parent Assessment Form	PCSI	1	68
Revised Life Orientation Test	LOT-R	1	72
Sports Concussion Assessment Tool 3	SCAT-3	1	72
Symptom Severity Scale	SSS	1	63
Vestibular Oculomotor Screening	VOMS	1	78

In addition, many studies used generalized questionnaires briefly ascertaining overall well-being^43,52,62,67^ and fatigue,^51,58,62,73^ presence of pain,^67^ past and current medical concerns,^42^ physical deficit or symptom checklists,^19,45,49,64^ both generalized and specific (e.g., headache, irritability, depression, memory problems, medication compliance, and other miscellaneous complications and symptoms). A handful of studies deployed similar generalized questionnaires, yet with a focus on reporting-by-proxy through the patient's caregiver.^69^

Where reported, these broader questions often existed either alongside or exclusive to generic questions encompassing: employment or return to work,^41,42,49,60^ household and leisure activities, or activities of daily living,^19,41,49,54,64,68^ mood,^42,67^ energy and sleep status,^67^ personal finance management,^41^ relationship status,^67^ subjective return to pre-injury or baseline status,^49^ travel and location,^41,62^ and lastly social context, and social activities and community participation.^41,62–64^ In pediatric populations, some generalized questionnaires sought information regarding the impact of injury or its sequelae on schooling and education,^49,68^ and play activities.^68^ One study with pediatric participants^54^ described measurement of daily cognitive rest and exertion by calculating a composite score of number of text messages sent, minutes of screen time and gaming, and minutes of reading and schoolwork.

Other domains, inclusive of those aforementioned, were assessed by one study as part of a structured interview addressing 17 broad domains including personal care, ambulation, home management, leisure, alcohol and drugs use, legal issues, and spirituality.^42^ One study^76^ differed from those previously reported in deploying a neuropsychological assessment battery (including standard verbally administered tests of attention, memory, working memory, processing speed, language, executive skills, and auditory-verbal adaptions of trail-making). Another study^71^ utilized informal oromotor and perpetual speech assessments as part of a wider speech-language battery. Weight status was included in one study with a longer duration of 36 weeks.^67^

One study, instead of directly asking for a subjective measure of travel, activities, or social and community participation, collected GPS-based activity data (MOVES Storylines) to quantify this measure objectively and indirectly.^61^ Similarly, another study^54^ utilized the device's onboard accelerometer to quantify step count as part of activity monitoring. A minority of studies sought to assess the access of further care as part of their technology-based follow-up assessment^19,49,69^—studies seeking this information were conducted solely in LMICs. One article describing the use of a telephone hotline for neurotrauma, by the nature of the technology differed greatly from others included by not reporting use of any outcome measures or other proforma for data collection.^53^

### Technology evaluation

Where technology was evaluated, most studies employed generalized questionnaires developed internally, and often used visual analog or 5- or 7-point Likert scales to gauge overall user satisfaction^61,62,65,69–73,77^ asking questions about acceptability, user friendliness, aesthetic, task difficulty, task appropriateness, and general impression of the technology. A smaller set of studies assessed technology feasibility^48,72,79^ (such as retrospectively analyzing those eligible for FUT enrollment, those consented, and those who completed all instances of assessment, among reasons for loss of contact where realized) and compliance,^72,73,77^ often which was calculated retrospectively rather than evaluated by the patient cohorts themselves, such as the use of a technology with respect to the investigator's target dose.^72^ Further, two studies reported anecdotal or qualitative feedback pertaining to the patient's experience in using the technology.^48,79^ One study asked participants to log daily unexpected, technology-related events such as errors as part of the evaluation process, and their perceived accuracy of the GPS-based activity data with respect to their actual activities.^61^ Only one study utilized an externally validated assessment of telehealth services, the Telehealth Usability Questionnaire (TUQ).^73^

## Discussion

The purpose of this review was to describe the breadth of technologies implemented for follow-up, and highlight the instruments deployed with respect to their successes for all-severity TBI in a global setting. Forty-two studies were retrieved that utilized FUTs for symptom surveillance and outcome data collection and described technologies that fell under broad categories of telephone-, SMS-, smartphone-, videoconference-based technologies among a small number of miscellaneous devices that may fit under a number of these categories.

Smartphones are widely recognized to be both well-positioned and well-suited for emotional,^73^ behavioral,^82^ and physical monitoring,^83^ particularly when applied to an EMA or experience sampling method (ESM) methodology. Traditional, face-to-face assessments conducted at infrequent intervals along the patient's journey of recovery rely upon retrospective self-reports that themselves are predisposed to recall bias,^84^ to which patients with TBI are believed to be more susceptible.^85^ This is further compounded by the numerous challenges patients with TBI face with cognitive impairment,^86^ poor memory,^87^ and impaired self-awareness.^88,89^ Smartphone and other FUT-based remote assessment, as mirrored in this review, show promise to quantify symptoms more accurately, and with respect to their temporal variability, otherwise uncaptured at a single time-point, and further facilitated in one's own natural environment.

The United Nations General Assembly in 2015 highlighted the impact technology-enabled breakthroughs have had in the health care sector, enabling greater numbers of people to have access to services otherwise out of reach or unaffordable.^90^ Although the implementation of technology to deliver follow-up is better than a complete absence of services, an informed understanding of the capabilities and technological fluency of the target population will be imperative for comprehensive and proper integration within standard practice. Several barriers to successful access of remote telehealth assessments and consultations have been identified in the literature and in recent WHO 2019 guidelines,^91^ including: disability such as hearing or cognitive impairment, lack of equipment, poor networking access and speeds, lack of organizational support, difficulty using the systems, security or privacy concerns, and unfamiliarity with technology.^91–94^ One qualitative study exploring non-participant views of a wider telehealth and telecare trial depicted that some patients were hesitant to adopt new approaches where existing face-to-face services were often highly valued.^93^ Patients with stigmatized health conditions may also possess additional concerns about the privacy of their information^91^ when handled digitally. Developing an awareness of the challenges faced by populations, particularly those most vulnerable such as the elderly and those recovering from the sequelae of injury, will ensure progress toward digital equality of service access, and mitigate the risk of bias or inaccurate data being introduced into technology-enhanced trials, registries, and injury surveillance campaigns.

Although the studies included often quantify their successes by compliance, adherence, and response rates, among broad and crude evaluations of these novel services, it is worth highlighting that any communication fostered by these technologies outside of predefined (and often sparsely scheduled) outpatient clinic meetings was appreciated and welcomed by both pediatric and adult patients and their caregivers, reflected by satisfaction rates reported in the studies included, and in part by strong compliance and return rates in the majority of FUT implementations in TBI. To our knowledge, no studies reported the use of a requirements elicitation survey or equivalent exercise in the design and development of FUTs, and thus this would be welcomed in future research in this area when examining what factors should be addressed and built into these services to improve patient compliance and satisfaction.

Further, the “successes” of technology-based follow-up delivery (of which one may consider to be derived from compliance, attrition, or response metrics) do not appear to be reported in a standardized format across the studies retrieved. As a result, conducting systematic comparisons between technology modalities, and across cohorts of varying severity and demographics, remains a challenge. Although the majority of studies attempted to quantify the success of their technology (Tables 1–5), such as comparing response rates versus prompts delivered, or the number of patients reached after an arbitrary number of contact attempts, further research or initiatives addressing this gap would serve favorably.

The compliance and satisfaction of clinicians remains key in developing technologies for integration into standard practice. It is not a new notion that neurosurgical services across the globe encounter heavy workloads and large patient numbers, especially in LMIC settings, where a disproportionate volume of cases must be handled by smaller specialist workforces when compared with their HIC colleagues.^95^ The benefits of asynchronous technologies thus become clear: enabling the clinician to review and act upon the data of their own accord, along with providing patients with the ability to self-report at their discretion, and at more frequent instances otherwise impossible to fulfill synchronously by clinical staff.

Wearable technologies such as smart watches, biometric monitors, and smart clothing may offer additional asynchronous sources of data and are slowly being introduced as medical technologies, although these were not identified in our review. Across medical disciplines, wearables have been demonstrated to enable real-time monitoring of vital signs, physical status, and physiological parameters as patients go about their daily lives.^96^ Although the literature describing wearables for TBI-afflicted individuals remains sparse,^97^ we clearly envision the role these technologies may play in enabling additional remote, data-driven approaches for post-TBI monitoring and early sequelae management.^98^

Lastly, it is of note that due to the global prevalence and availability of mobile phone or telephone services, many studies screened often briefly stated in their reports the use of a telephone for follow-up practice; however, they excluded a deeper insight, evaluation, or formal assessment of the utility of the remote technology for conducting the act of said follow-up itself. In this respect, due diligence was exercised by the authors across the screening stages.

This scoping review serves as a foundation for the application of technology in follow-up and outcome data collection. Herein, we propose a number of recommendations for future research and practice.

First, from this review, there appears to be grounds for further research exploring, and perhaps refining, what outcome measures are deployed, and at what intervals assessments are conducted. Table 6 reveals a broad range of outcome measures used in the assessment of patients with TBI, some measures of which were deployed in multiples. To ensure technology functions optimally and simultaneously for both patients and clinicians, a balance must be struck between the richness of assessments and the efforts required on the patient's behalf in quantifying their health and well-being at a distance. Further, it would be beneficial for future research to assess the validity of outcome measures when delivered sequentially in a single instance, and namely whether delivering multiple outcome measures introduces confounding effects, including when delivered remotely and without the option of clinical assistance or clarification. Similarly, when they have been applied to FUT-led research it would be beneficial to explore and address the validities of outcome measures that were initially designed to be deployed on a face-to-face basis. We believe that there may be assumptions to challenge regarding whether a party perceived an outcome measure could be successfully delivered by a technological medium with ease, that the validities of such a measure must transfer simultaneously. As such, we encourage further efforts into FUT-led research practices themselves.

Second, a wide variety of outcome measures and instruments have been deployed by technology for remote assessment. Although beyond the scope of this review, it may be useful for further research to retrospectively address the validity of measures deployed, often designed for in-person use, with respect to their adaption for FUT deployment. Lastly, further investigation may be warranted to assess the effects on outcome measure validity in studies deploying multiple patient-reported outcome measures at single or close-together time-points. In addition, for this research investigators may wish to examine whether these instruments when delivered individually or as sets remotely can continue to accurately quantify outcomes from the acute to long-term stages of all-severity TBI and where these technologies may assist in facilitating data collection for common data elements as part of large-scale research efforts.

### Strengths and limitations

To our knowledge, this review is among the first to map the current global evidence base of technologies deployed to augment traditional modes of in-person follow-up. A broad and comprehensive search across five electronic databases was conducted, and as such this review serves as a strong foundation for understanding the use-cases of technology-based follow-up for TBI in a global setting. Although the number of studies retrieved is relatively small considering our eligibility criteria (FUTs employed in a global context for all-severity TBI in pediatric and adult cohorts), we present a particular slice of the evidence base in which included articles have a primary focus of exploring FUT deployment.

In the context of future research, we trust this review will serve further as a comprehensive frame of reference for those wishing to apply FUTs in clinical practice or research. However, owing to a sheer number of terms describing global health, technologies, and their implementation, outcome measures, and follow-up, some published articles that may have met the predefined inclusion criteria could have been inadvertently excluded from this review. Further, we acknowledge limitations of our retrieved articles with respect to our eligibility criteria; we are aware of a number of large, high-profile injury surveillance studies, such as those utilizing the U.S. TBI Model Systems (TBIMS) database,^99–102^ that were not included, despite using FUTs in their research, as a description or assessment of FUTs was not the primary focus of such works. Similarly, by the nature of global health and technological reports, which may not always be confined to health journals and/or databases, or always depicted in the English language, we are aware of the limitations in the literature retrieved, and of the literature that may exist in alternate academic or commercial domains and mediums. Considering these limitations, and with an understanding of the parameters of FUTs afforded by this review, a further systematic review as the evidence base evolves, inclusive of gray literature, non-English publications, and articles utilizing FUTs but not necessarily as their primary aim, is recommended.

## Conclusions

Our review has demonstrated that the evidence base surrounding FUTs remains in its infancy, particularly with respect to recruiting large patient cohorts, conducting a formal technology assessment, and the representation of research outside high-income settings, respectively. Of the use-cases described, incorporating technologies, both asynchronous and synchronous in nature, may leverage a clinician's abilities in gaining insights of the patient's well-being from discharge and beyond between traditional and often sparsely scheduled face-to-face appointments. FUTs may additionally serve to provide a more precise picture of the status of the patient with TBI through their ability to collect data at time-points closer in proximity to in-person follow-up, harmonious with the WHO's adjuration and promotion of outcome data collection and injury surveillance in the reduction of global TBI burden. Further systematic analyses may prove useful in empirically quantifying the utility, acceptability, feasibility, and costs of each technology modality in neurotrauma practice. Future research may wish to characterize the challenges of implementing, sustaining, and adhering to these novel systems from the perspectives of patients, their proxies, and physicians.

## Supplementary Material

Supplemental data

Supplemental data
