# Supplementary material for: Remote Follow-Up Technologies in Traumatic Brain Injury: A Scoping Review
Source: J Neurotrauma. 2022 Sep 29;39(19-20):1289–317. doi: 10.1089/neu.2022.0138 (PMC9529313; doi:10.1089/neu.2022.0138)
Supplement: Supplemental data [file Supp_TableS2.docx]

**Example search strategy for OVID Medline**

| 1. (sms or short messag* service or texting or text messag* or messag* or mms or multimedia messag* service or videoconferenc*).mp. [mp=title, abstract, original title, name of substance word, subject heading word, floating sub-heading word, keyword heading word, organism supplementary concept word, protocol supplementary concept word, rare disease supplementary concept word, unique identifier, synonyms]  2. (cell-phone* or cell*phone* or cellular phone* or telephon* or hand-phone* or hand phone* or smartphone*).mp. [mp=title, abstract, original title, name of substance word, subject heading word, floating sub-heading word, keyword heading word, organism supplementary concept word, protocol supplementary concept word, rare disease supplementary concept word, unique identifier, synonyms]  3. (telemed* or tele*med* or tele-med* or teleconsult* or telesurg* or telehealth or remote consult* or mhealth or mobile health or ehealth).mp. [mp=title, abstract, original title, name of substance word, subject heading word, floating sub-heading word, keyword heading word, organism supplementary concept word, protocol supplementary concept word, rare disease supplementary concept word, unique identifier, synonyms]  4. exp Telecommunications/  5. exp Mobile Applications/  6. exp Web Browser/  7. exp Internet/  8. exp Outcome Assessment, Health Care/  9. exp Patient Generated Health Data/  10. exp Surveys/ and Questionnaires/  11. exp Patient Reported Outcome Measures/  12. exp Ecological Momentary Assessment/  13. (ecologic* momentary assessment* or community assessment*).mp. [mp=title, abstract, original title, name of substance word, subject heading word, floating sub-heading word, keyword heading word, organism supplementary concept word, protocol supplementary concept word, rare disease supplementary concept word, unique identifier, synonyms]  14. outcome measure*.mp. [mp=title, abstract, original title, name of substance word, subject heading word, floating sub-heading word, keyword heading word, organism supplementary concept word, protocol supplementary concept word, rare disease supplementary concept word, unique identifier, synonyms]  15. (TBI or traumatic brain injur* or brain injur* or head injur* or concuss* or brain traum* or head traum*).mp. [mp=title, abstract, original title, name of substance word, subject heading word, floating sub-heading word, keyword heading word, organism supplementary concept word, protocol supplementary concept word, rare disease supplementary concept word, unique identifier, synonyms]  16. exp Craniocerebral Trauma/  17. 1 or 2 or 3 or 4 or 5 or 6 or 7  18. 8 or 9 or 10 or 11 or 12 or 13 or 14  19. 15 or 16  20. 17 and 18 and 19 |
| --- |
