# Supplementary material for: Remote Follow-Up Technologies in Traumatic Brain Injury: A Scoping Review
Source: J Neurotrauma. 2022 Sep 29;39(19-20):1289–317. doi: 10.1089/neu.2022.0138 (PMC9529313; doi:10.1089/neu.2022.0138)
Supplement: Supplemental data [file Supp_TableS1.docx]

**Data characterisation proforma**

| 1. **Authorship characteristics** | | |
| --- | --- | --- |
| **Variable** | **Category** | **Explanation** |
| Author(s) | ☐ _____________________ | Please list. |
| Year of Publication | ☐ _____________________ | Please state. |
| Country | ☐ _____________________ | Please state the setting where the study took place. |

| 1. **General Study Characteristics** | | |
| --- | --- | --- |
| **Variable** | **Category** | **Explanation** |
| Study design | ☐ Descriptive  ☐ Experimental  ☐ Observational  ☐ Qualitative  ☐ Mixed Methods | Please select. |
| Objective(s) | ☐ _____________________ | Please briefly describe. |
| Population size | ☐ _____________________ | Please state. |
| Population age / gender | ☐ _____________________ | Please state a range of ages / gender. |
| Population socio-economic status | ☐ _____________________  ☐ Not reported | Please describe any details pertaining to socio-economic status, e.g. income, level of education, type of occupation military, student, sports etc. |
| Data collection setting | ☐ Home/community  ☐ Primary care setting  ☐ Secondary care setting  ☐ Tertiary care setting  ☐ Other:  _______________________  ☐ Not reported | Please select where patient primarily found at time of data collection.  For all data collection settings follow-up must still be conducted remotely with the clinical team responsible for their care.  Primary care settings may include a general practice or small community-based clinic.  Secondary care settings include community hospitals, rehabilitation centres, outpatient clinics. |

| 1. **Description of neurotrauma** | | |
| --- | --- | --- |
| **Variable** | **Category** | **Explanation** |
| Severity of traumatic brain injury | ☐ Severe  ☐ Moderate  ☐ Mild / Concussion  ☐ Other:  _______________________ | Please select. Severity according to Glasgow Coma Scale:  *GCS 13 – 15 = Mild*  *GCS 9 – 12 = Modearate*  *GCS 3 – 8 = Severe* |
| Injury characteristics | ☐ _____________________  ☐ Not reported | Please state if reported. |

| 1. **Description of follow-up technology utilised** | | |
| --- | --- | --- |
| **Variable** | **Category** | **Explanation** |
| Use-case description | ☐ _____________________ | Please briefly describe. |
| Format of technology | ☐ SMS / text-message  ☐ Telephone call  ☐ Mobile application  ☐ Web application  ☐ Videoconferencing  ☐ Other:  _______________________ | Please select all that apply. |
| Device(s) used to facilitate technology | ☐ Mobile phone  ☐ Telephone  ☐ Smartphone  ☐ Home computer  ☐ Wearable technology  ☐ Videoconferencing  ☐ Other:  _______________________ | Please state if reported. |
| Outcome measure(s) / assessment(s) / instrument(s) employed in study | ☐ _____________________ | Please briefly describe. |
| Follow-up duration | ☐ _____________________ | Please state. |
| Iterations / number of implementations of the follow-up technology | ☐ _____________________  ☐ Not reported | Please describe, eg. how many full assessments handled remotely and details. |

| 1. **Major findings** | | |
| --- | --- | --- |
| **Variable** | **Category** | **Explanation** |
| Major findings | ☐ _____________________  _______________________  _______________________  _______________________ | Please describe. |
| Major challenges | ☐ _____________________  _______________________  _______________________ | Please describe. |
